# Cellular Architecture Regulates Collective Calcium Signaling and Cell Contractility

**DOI:** 10.1371/journal.pcbi.1004955

**Published:** 2016-05-19

**Authors:** Jian Sun, James B. Hoying, Pierre A. Deymier, Donna D. Zhang, Pak Kin Wong

**Affiliations:** 1 Department of Mechanical Science and Engineering, University of Illinois at Urbana-Champaign, Urbana, Illinois, United States of America; 2 Department of Aerospace and Mechanical Engineering, The University of Arizona, Tucson, Arizona, United States of America; 3 Cardiovascular Innovation Institute, University of Louisville & Jewish Hospital, Louisville, Kentucky, United States of America; 4 Material Science and Engineering Department, The University of Arizona, Tucson, Arizona, United States of America; 5 Department of Pharmacology and Toxicology, The University of Arizona, Tucson, Arizona, United States of America; 6 Departments of Biomedical Engineering, Mechanical Engineering, and Surgery, The Pennsylvania State University, University Park, Pennsylvania, United States of America; University of Virginia, UNITED STATES

## Abstract

A key feature of multicellular systems is the ability of cells to function collectively in response to external stimuli. However, the mechanisms of intercellular cell signaling and their functional implications in diverse vascular structures are poorly understood. Using a combination of computational modeling and plasma lithography micropatterning, we investigate the roles of structural arrangement of endothelial cells in collective calcium signaling and cell contractility. Under histamine stimulation, endothelial cells in self-assembled and microengineered networks, but not individual cells and monolayers, exhibit calcium oscillations. Micropatterning, pharmacological inhibition, and computational modeling reveal that the calcium oscillation depends on the number of neighboring cells coupled via gap junctional intercellular communication, providing a mechanistic basis of the architecture-dependent calcium signaling. Furthermore, the calcium oscillation attenuates the histamine-induced cytoskeletal reorganization and cell contraction, resulting in differential cell responses in an architecture-dependent manner. Taken together, our results suggest that endothelial cells can sense and respond to chemical stimuli according to the vascular architecture via collective calcium signaling.

## Author Summary

Cells can sense and respond to their microenvironments collectively to yield high-order complex multicellular behaviors. Despite its fundamental importance in cell physiology, the emerging mechanisms that drive collective cell signaling and their functional implications in diverse cellular structures, e.g., as seen in various vascular architectures, remain poorly understood. These collective cell behaviors are often counterintuitive and cannot be understood by extrapolating the behaviors of individual cells. Using a combination of microscale cell patterning and computational modeling, we elucidate the architecture-dependent calcium signaling and contractility in endothelial cells via gap junction mediated calcium oscillation in an architecture-dependent manner. As calcium is one of the most important second messengers, collective calcium signaling may serve as a broad mechanism in regulating various cell functions. More importantly, our finding highlights a relatively unexplored mechanism of cellular regulation via cellular architectures.

## Introduction

The calcium ion is a universal second messenger mediating a wide range of dynamic cell functions, such as exocytosis, contraction, transcription, and proliferation [1–3]. These biological processes that span time scales from microseconds to hours are regulated by diverse calcium signaling mechanisms. A hallmark of calcium signaling is the transient pulsing dynamics of cytosolic calcium concentration. The calcium dynamics are actively controlled by calcium release from intracellular stores and calcium influx from extracellular media, outflow by pumps and exchangers, and calcium binding proteins, which fine-tune the amplitude and duration of calcium pulses to regulate physiological functions. Calcium oscillation, for instance, can sensitize signal detection at low agonist concentration and its frequency can also encode additional information that selectively controls transcription factor activity [4–6].

The calcium dynamics are regulated by multiple intracellular calcium processing mechanisms, such as non-selective cation channels, voltage-dependent calcium channels, store-operated calcium channels, calcium-induced calcium release, and phospholipase C mediated inositol 1,4,5-trisphosphate activity [3]. Intercellular collective calcium signaling mechanisms can also modulate the calcium dynamics. In particular, gap junctional intercellular communication (GJIC) mediates coupling currents between neighboring cells triggered by an imbalance in the membrane potential. GJIC also allows calcium diffusion driven by a difference in the cytosolic calcium concentration [7, 8]. For instance, calcium waves can propagate among endothelial cells, and intercellular calcium signaling provides a robust mechanism for mechanosensing in endothelial networks [9, 10]. Three-dimensional coupling of β-cells can lead to robust synchronized calcium oscillations and insulin secretion at elevated glucose levels [11]. GJIC also regulates asymmetric neuronal fates [12] and controls collective chemosensing to generate synchronized and coordinated responses under adenosine triphosphate (ATP) stimulation [13, 14]. Physiologically, histamine is known to induce calcium signaling and cell contraction in endothelial cells. Interestingly, histamine-induced permeability is confined to venules rather than capillaries [15], suggesting an architecture dependence on the response to histamine. Nevertheless, the effects of structural arrangement, as seen in diverse vascular structures, on calcium signaling and their functional implications are poorly understood.

In this study, we investigate architecture-dependent collective calcium signaling in human umbilical vein endothelial cells (HUVEC). Self-assembled capillary-like networks [16] and microengineered hexagonal cell networks [17] are created to evaluate the effects of cellular architecture on calcium signaling. Endothelial cell networks of arbitrary shapes and widths can be created by plasma lithography cell patterning [18]. The method has been demonstrated for investigating the effects of geometric confinements on various biological systems [19–22]. Histamine-induced calcium oscillations in the cell networks are compared with individual cells and monolayers to investigate the architecture dependence. Along with micropatterning and pharmacological inhibition, a computational model is developed to elucidate the effects of the number of neighboring cells on collective calcium signaling. The cytoskeletal reorganization and cell contraction induced by histamine are correlated with the calcium oscillation to study the function of collective calcium signaling in different cellular architectures.

## Results

### Architecture-dependent collective calcium signaling

**Fig 1:**
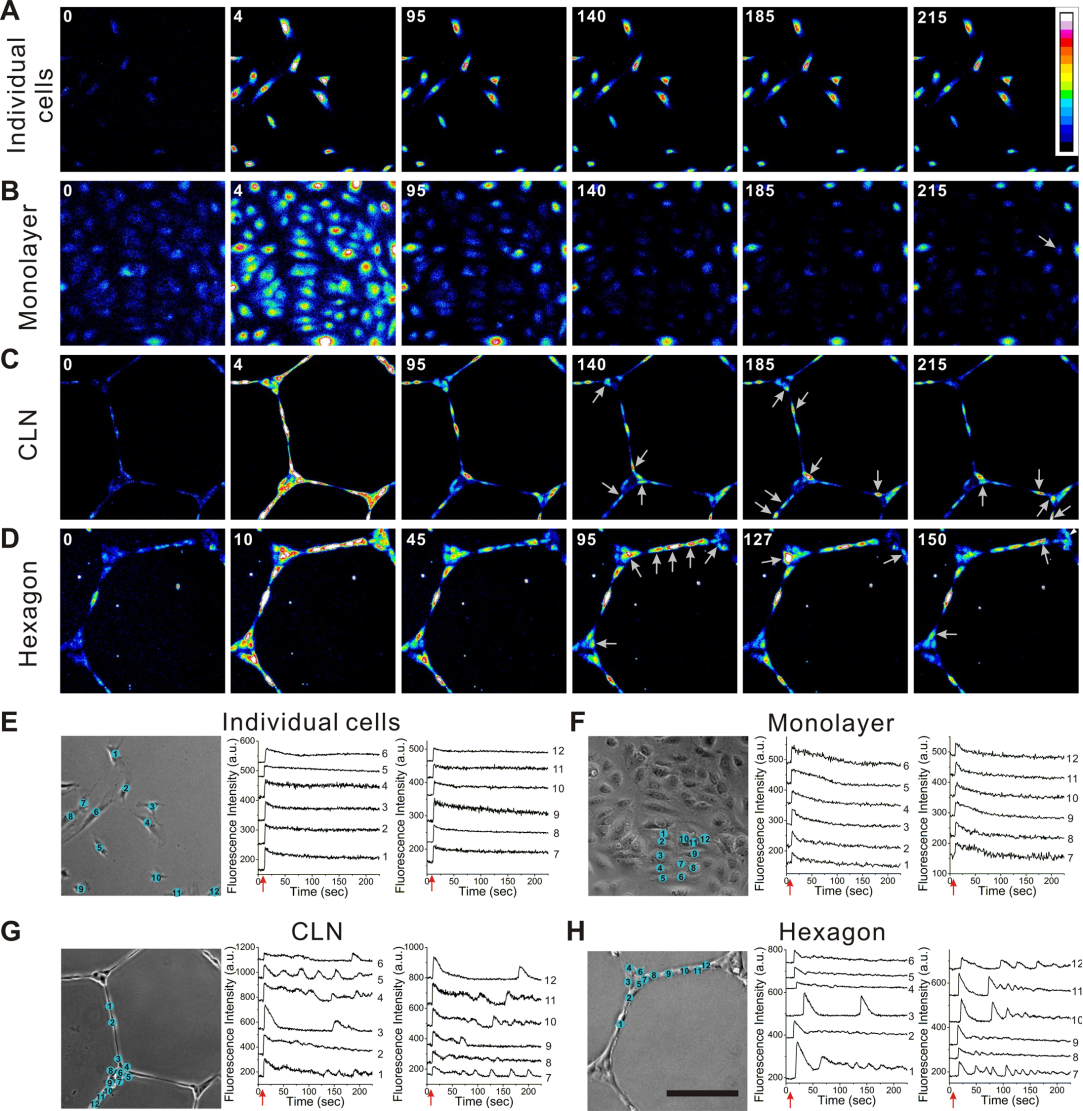
Calcium signaling in HUVEC depends on the cellular architecture. (A–D) Histamine-induced calcium dynamics in (A) individual cells (100 cells/mm^2^), (B) cells in monolayers (800 cells/mm^2^), (C) capillary-like networks (CLN), and (D) microengineered hexagonal cell networks (hexagon). The numbers at the upper left corners indicate the time in seconds after histamine treatment. White arrows indicate cells exhibiting calcium oscillations. (E–H) Calcium dynamics in 12 selected cells in different configurations. Red arrows indicate the time of histamine addition. The calcium response curves were shifted vertically for clarity. Images are representative from three to eight independent experiments (n = 6 for individual cells; n = 6 for monolayer; n = 3 for CLN; n = 8 for hexagon). Scale bar, 200 μm. Random regions of interest at 405 μm×405 μm were chosen to ensure enough cells for representing the calcium signaling in various cell architectures. For individual cells, cells were numbered randomly. For monolayers and cell networks, cells were numbered to illustrate the spatial locations.

To explore the effects of cellular architecture, histamine-induced calcium signaling was monitored in individual endothelial cells, monolayers, self-assembled capillary-like networks, and plasma lithography microengineered cell networks (Fig 1). Histamine treatment (5 μM) induced a rapid increase followed by a slow decay of cytosolic calcium concentration in individual cells (Fig 1A and 1E and S1 Video) and in monolayers (Fig 1B and 1F and S2 Video). Mean decay rate, the reciprocal of half decay time, was 0.0087±0.0003 sec^-1^ for individual cells and 0.0180±0.0018 sec^-1^ for cells in monolayer. Only a small fraction of cells (~8%) exhibited cytosolic calcium oscillations. This observation is consistent with previous reports that histamine does not induce calcium oscillations in human endothelial cells at high concentrations (over 3 μM) [23]. Surprisingly, the cells in self-assembled capillary-like networks exhibited short initial calcium pulses with a rapid mean decay rate of 0.0380±0.0032 sec^-1^. The decay rate was significantly faster than the values in individual cells and monolayers. Importantly, calcium oscillations occurred in a much larger portion (~55%) of cells in self-assembled capillary-like networks (Fig 1C and 1G and S3 Video). The oscillations displayed irregular patterns and did not show any apparent synchronization between neighboring cells.

**Fig 2:**
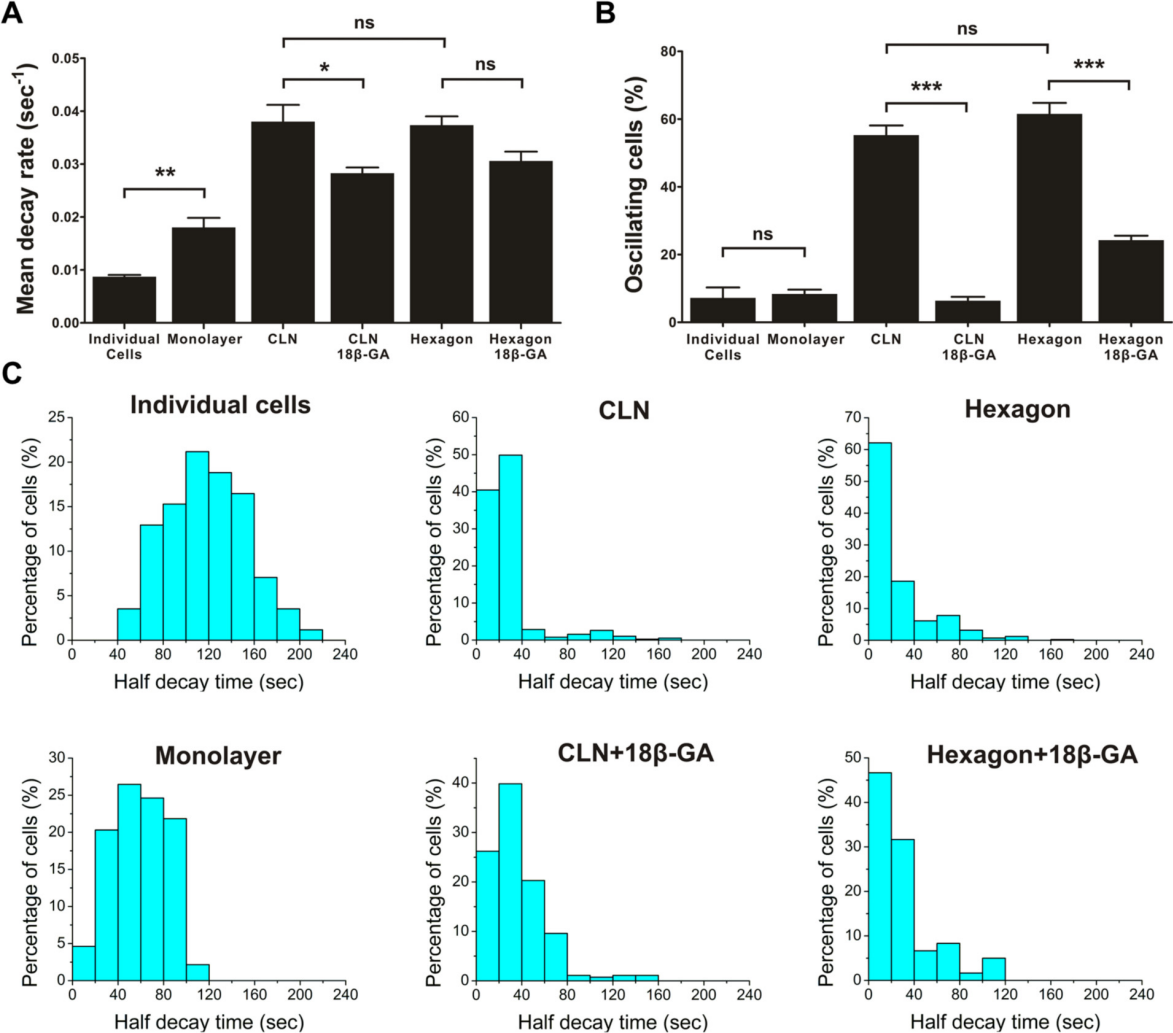
Architecture-dependent calcium signaling is mediated through gap junctions. (A-B) Quantification of the mean decay rate of the initial calcium spike and calcium oscillation occurrence rate in various configurations in the presence or absence of 18β-GA, a gap junction blocker. Data represent mean ± s.e.m. (Bonferroni's multiple comparison test; ns, not significant; *, P<0.05; **, P<0.01; ***, P<0.001; n = 6 for individual cells; n = 3 for CLN; n = 3 for CLN with 18β-GA; n = 8 for hexagon; n = 3 for hexagon with 18β-GA). (C) Histograms of the half decay times of the initial calcium spikes. Total cells analyzed were 256, 2776, 466, 377, 884, and 324 for individual cells, monolayer, CLN, CLN with 18β-GA, hexagon, and hexagon with 18β-GA respectively.

To confirm the calcium oscillation was triggered by the cellular architecture, instead of the physical or biochemical properties of the extracellular matrix, endothelial cells were patterned into hexagonal cell networks on polystyrene by plasma lithography (S1 Fig). Similar to capillary-like networks, calcium oscillations occurred in a large portion (~61%) of cells and displayed irregular calcium patterns without apparent synchronization (Fig 1D and 1H). The calcium pulses of cells in hexagonal cell networks also had a rapid mean decay rate of 0.0374±0.0017 sec^-1^. There is no significant difference between capillary-like networks and hexagonal cell networks for the mean decay rate (Fig 2A) and the calcium oscillation occurrence rate (Fig 2B). The cell response time, calcium increase rate, and amplitude did not depend on the cellular architecture. These results indicate that the calcium dynamics of endothelial cells, in particular the occurrence of calcium oscillation, depends on the cellular architecture.

### Architecture-dependent calcium signaling is GJIC dependent

Gap junctions, which electrically and chemically couple endothelial cells, play significant roles in intercellular communication and cell functions [24, 25]. To decipher the roles of GJIC in the observed architecture-dependent calcium signaling, endothelial cells in capillary-like networks and hexagonal cell networks were pretreated with a gap junction blocker, 18-β-glycyrrhetinic acid (18β-GA, 40 μM) [26, 27]. With histamine treatment, cytosolic calcium levels in 18β-GA treated cells increased abruptly and gradually decreased to the basal level, similar to individual cells and monolayers (S2 Fig). The mean decay rate was 0.0283±0.0011 sec^-1^ and 0.0306±0.0018 sec^-1^ for capillary-like networks and hexagonal cell networks, respectively (Fig 2A). The calcium oscillation occurrence rates were reduced significantly in both capillary-like networks and hexagonal cell networks (Fig 2B). The distributions of the half decay time of calcium pulses for various cell patterns further indicated that the portion of cells with a prolonged calcium response increased with 18β-GA treatment (Fig 2C). These results suggest the architecture-dependent calcium oscillation is GJIC dependent.

### Computational analysis of pluricellular calcium dynamics

**Fig 3:**
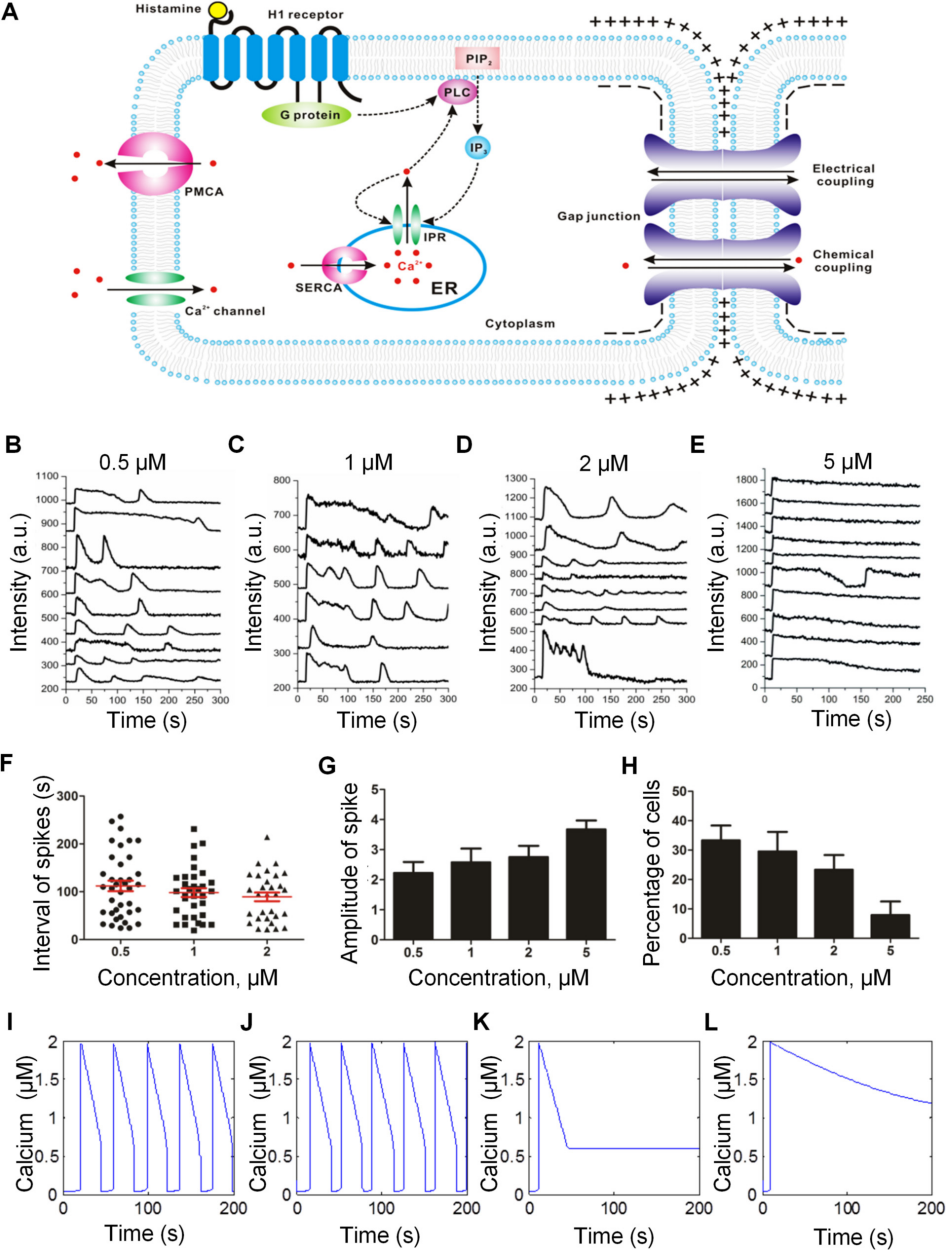
Computational modeling of calcium signaling. (A) Histamine-induced calcium signaling in multicellular systems. Histamine induces the level of inositol 1,4,5-trisphosphate (IP_3_) via histamine H1 receptor and phospholipase C (PLC) signaling. Sarco/endoplasmic reticulum Ca^2+^-ATPase (SERCA) and IP_3_ receptor (IPR) regulate calcium fluxes between the cytosol and the endoplasmic reticulum (ER). Plasma membrane Ca^2+^-ATPase (PMCA) and calcium channels transport calcium between the cytosol and the extracellular space. With GJIC, the cytosolic calcium concentration can be perturbed by the coupling current due to an imbalance in membrane potential and calcium diffusion due to a concentration gradient. (B-E) Representative calcium dynamics of individual endothelial cells under different concentrations of histamine measured experimentally. The calcium response curves were shifted vertically for clarity. (F) Intervals between the first two spikes in cells exhibiting calcium oscillation. Cells treated with 5 μM histamine were not included due to the small number of cells that exhibit calcium oscillations. (G) Amplitude of calcium pulses at different histamine concentrations. (H) The percentage of cells exhibiting calcium oscillations at different histamine concentrations. Data are representative from three independent experiments. For each independent experiment, ~10 individual cells were analyzed. (I-L) Calcium dynamics of individual endothelial cells under different concentrations of histamine calculated computationally. Parameters applied in the computational models were (I) *K* = 1 s^-1^ and *V*_*m3*_ = 300 s^-1^; (J) *K* = 0.75 s^-1^ and *V*_*m3*_ = 400 s^-1^; (K) *K* = 0.5 s^-1^ and *V*_*m3*_ = 500 s^-1^; (L) *K* = 0.1 s^-1^ and *V*_*m3*_ = 600 s^-1^ to show the effects of increasing histamine concentration.

We investigate the mechanisms of architecture-dependent calcium oscillation using a computational model [8, 28]. This model describes calcium fluxes from intracellular stores and extracellular media to the cytosol while simultaneously considering intercellular fluxes due to electrical and biochemical coupling between neighboring cells via GJIC (Fig 3A). The calcium fluxes are regulated by the cytosolic calcium concentration through multiple feedback loops, which capture the dynamics of plasma membrane Ca^2+^ ATPase, calcium channels, endoplasmic reticulum uptake, and inositol 1,4,5-trisphosphate activity. A detailed description of the computational model is provided in S1 Text. The model generated calcium pulses and calcium-induced calcium release as observed in endothelial cells. Experiments were first performed to validate the accuracy of the model. In the experiment, endothelial cells were treated with different concentrations of histamine (Fig 3B–3E). The interval between pulses, amplitude of pulses and the calcium oscillation occurrence rate are summarized in Fig 3F, 3G and 3H. The computational model successfully predicted the weak dependence of the interval between pulses on histamine concentration (Fig 3F, 3I, and 3J). The amplitude of the pulses was independent of histamine concentration (Fig 3G). Most importantly, the model successfully captured the prolonged calcium response and low calcium oscillation occurrence rate for cells treated with high (5 μM) concentrations of histamine (Fig 3H, 3K and 3L). The computational model, therefore, captured the essence of calcium dynamics observed in our experiment, supporting its use for investigating the architecture-dependent calcium signaling.

**Fig 4:**
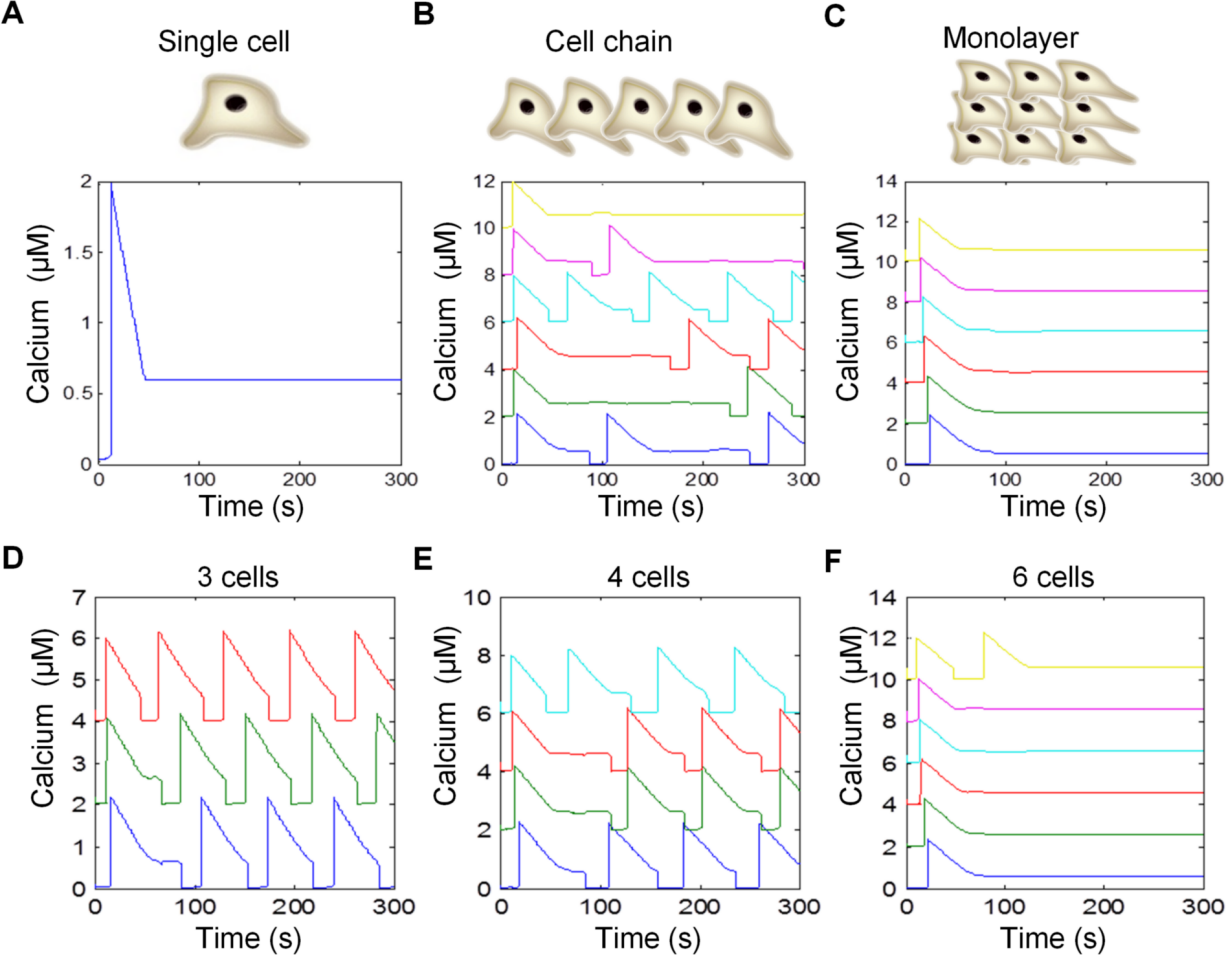
Computational modeling of architecture-dependent calcium signaling. (A–C) Effects of cellular architectures on calcium signaling in (A) a single cell, (B) 9 cells connected linearly as a chain with periodic boundary conditions, and (C) 9 cells connected in a monolayer with periodic boundary conditions. The calcium dynamics of six cells were shown for clarity. (D–F) Calcium dynamics for (D) 3, (E) 4, and (F) 6 coupled cells. The data were shifted vertically for clarity.

The computational model was applied to study the effects of calcium diffusion and coupling current on the calcium dynamics. Individual cells did not exhibit calcium oscillations in the simulation (Fig 4A). When two cells were electrically coupled, a coupling current was generated due to a small difference in the membrane potentials, which tended to equalize the membrane potentials. To stabilize the membrane potential, the coupling current was compensated by other currents, including the calcium current, which perturbed the cytosolic calcium concentration and induced irregular, unsynchronized calcium oscillations (S3 Fig). The electrical coupling formed the basis of calcium oscillation in coupled cells [8, 28]. When multiple cells were connected as a linear chain (i.e., each cell was coupled to two immediate neighbors), the model predicted that most cells would exhibit calcium oscillation. Fig 4B shows the behavior of nine cells connected linearly. Similar to the capillary-like and hexagonal cell networks, irregular patterns of calcium pulses were observed and the oscillation did not display apparent synchronization between neighboring cells. Remarkably, a different behavior emerged when the number of coupled cells increased. When the cells were connected in an array and coupled to each other (e.g., monolayer), the calcium oscillations vanished in all of the cells. Fig 4C shows the calcium dynamics when nine cells are coupled. The cells exhibited prolonged calcium responses and did not display any oscillation, similar to the experimental observation. Thus, the computational simulation captures the architecture-dependent calcium signaling observed in the experiment.

Examining the computational model provides insights into the mechanism of the architecture-dependent calcium signaling. In particular, the intracellular calcium dynamics driven by the calcium-induced calcium release were highly sensitive to the cytosolic calcium concentration. The membrane potential and coupling current created the oscillations in coupled cells. On the other hand, the intercellular diffusion of calcium could stabilize the coupled cells by maintaining the cytosolic calcium at the steady state value. This stabilizing capability increased with the number of cells coupled. Fig 4D, 4E and 4F shows the cytosolic calcium dynamics when 3, 4, and 6 cells were coupled. The calcium oscillation occurrence rate decreased as the number of cells increased. Oscillations were not observed when seven or more cells were coupled. Similar to the gap junction blocker experiment, eliminating the coupling in the computational model suppressed calcium oscillations and resumed the calcium dynamics of individual cells.

### Collective calcium signaling depends on the number of neighboring cells

**Fig 5:**
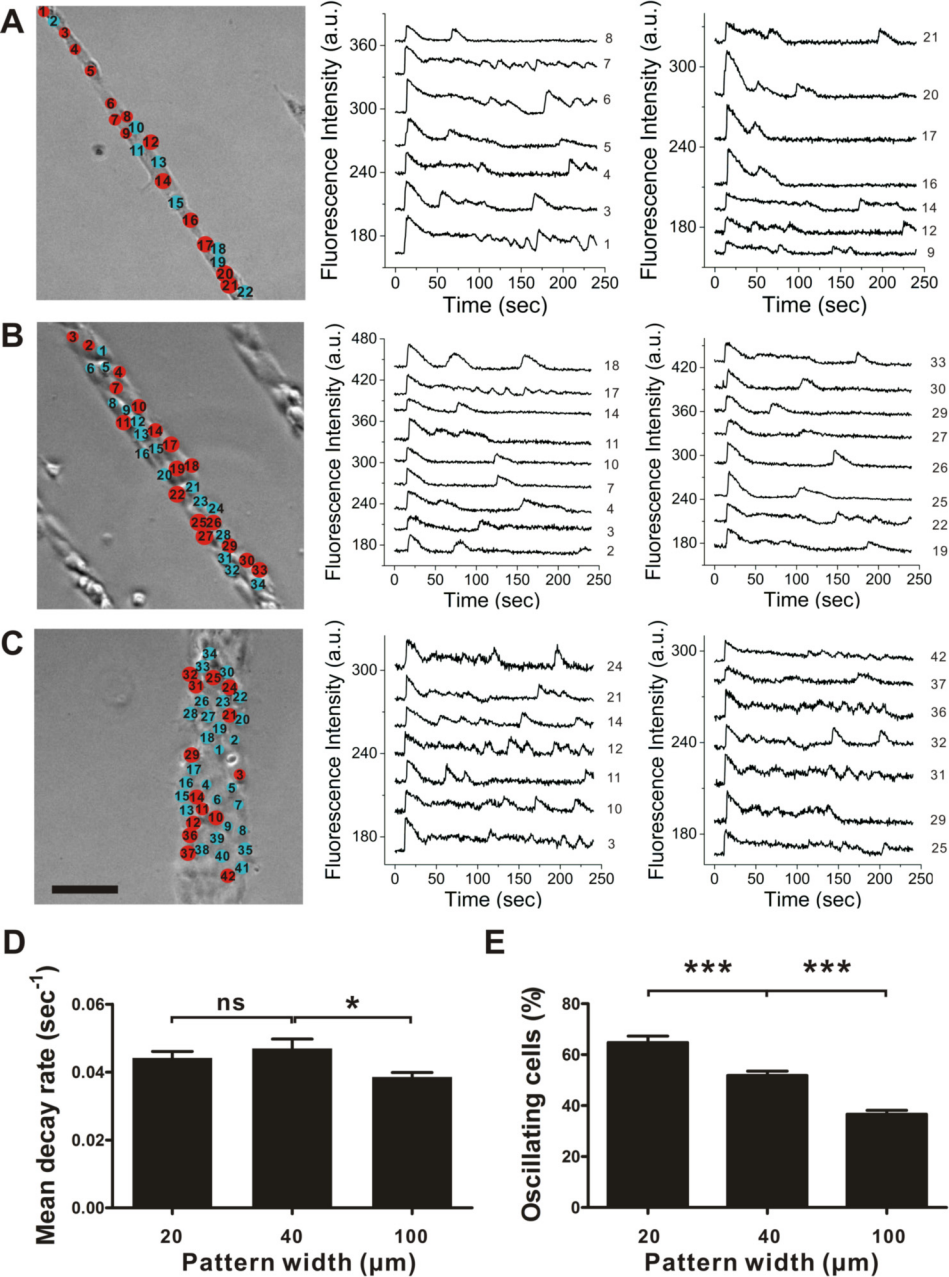
Collective calcium signaling depends on the number of coupled cells in linear networks. (A–C) Histamine-induced calcium oscillations in linear cell networks with the width of (A) 20 μm, (B) 40 μm and (C) 100 μm. Scale bar, 100 μm. Red circles indicate the oscillating cells. The calcium response curves were shifted vertically for clarity. (D-E) Quantification of the mean decay rate and calcium oscillation occurrence rate in linear cell networks with different widths (n = 8; 233 cells for 20 μm; 432 cells for 40 μm; 806 cells for 100 μm; Bonferroni's multiple comparison test; ns, not significant; *, P<0.05; ***, P<0.001).

The computational model predicts that the calcium dynamics are sensitive to the number of cells coupled. To test the effects of the number of coupled cells on intercellular calcium dynamics experimentally, linear cell networks with various widths were designed and patterned via plasma lithography (Fig 5). In particular, linear cell networks with the width of a single cell to multiple cells (20–100 μm) were created. The average number of neighboring cells increased from 2 to 8 in these linear networks. Upon histamine stimulation, the cells in linear networks displayed cytosolic calcium pulses with different decay rates (Fig 5A, 5B and 5C). The mean decay rate decreased as the line width increased from 40 μm to 100 μm (Fig 5D). Importantly, the calcium oscillation occurrence rate decreased gradually as the width of linear patterns increased from 20 μm to 100 μm (Fig 5E). In addition, cells at the edge (not surrounded on all sides) are more likely to undergo calcium oscillations than cells at the inner region (surrounded on all sides) at the presence of 5 μM histamine for capillary-like networks, hexagonal cell networks and 100 μm wide linear cell networks (S4 Fig). Therefore, both experimental and computational models support that the calcium oscillation occurrence rate negatively correlated with the number of neighboring cells in the network.

### Collective calcium signaling regulates cytoskeletal reorganization

**Fig 6:**
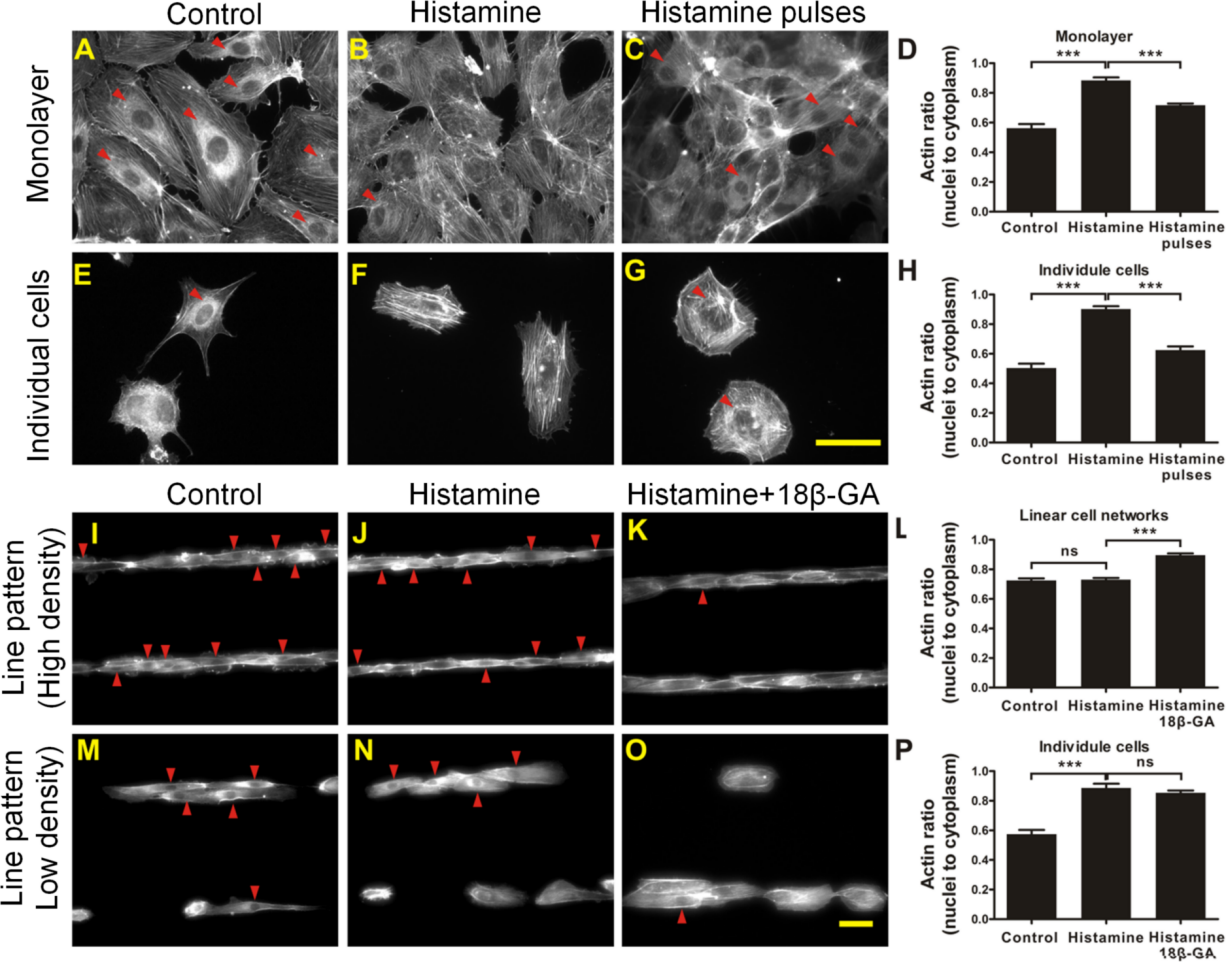
Actin cytoskeleton of cells in different configurations after histamine treatment. (A–C) Monolayers. (E–G) Individual cells. (I–K) Linear cell networks with high cell density. (M–O) Linear cell networks with low cell density. Cells were stained with Alexa Fluor® 555 Phalloidin for F-actin. Results are representative of 5 independent experiments. (D, H, L and P) Quantitative analysis of the actin ratio (nucleus to cytoplasm) in (D) monolayers, (H) individual cells, (L) linear cell networks, and (P) individual cells in linear pattern with low cell density (Bonferroni's multiple comparison test; ns, not significant; ***, P<0.001; n = 5). Red arrows indicate cells with actin ratio of nucleus to cytoplasm below 0.7. Scale bars, 50 μm.

Histamine mediates changes in actin cytoskeleton, induces intercellular gap formation, increases endothelial permeability, and causes endothelial barrier dysfunction through calcium-mediated cell contraction [29–32]. The effects of histamine on the actin cytoskeleton were investigated to explore the potential impact of collective calcium signaling on physiological cell functions. Before histamine treatment, dense F-actin structures surrounding the nuclei (but not in the nuclei) were observed in individual cells and monolayers (Fig 6A and 6E). The fluorescence intensity in the nucleus region was significantly lower than the values in the cytoplasm. After histamine treatment for 5 min, the F-actin structures reorganized and stress fibers formed across the cells, including the nucleus region (Fig 6B and 6F). With the stress fiber formation after cytoskeletal reorganization, the fluorescence intensity in the nucleus region was similar to the values in the cytoplasm. The ratio of fluorescence intensities between nucleus and cytoplasm was therefore employed to quantify the actin distribution before and after histamine treatment. Cytoskeletal reorganization was defined quantitatively when the ratio of fluorescence intensities was above 0.7. Red arrows in Fig 6 indicate cells with dense F-actin structures surrounding the nuclei (i.e., no cytoskeletal reorganization).

To investigate the influence of collective calcium signaling on cytoskeletal reorganization in cell networks, endothelial cells in linear networks were exposed to histamine for 5 minutes. Unlike individual cells and monolayers, the dense F-actin structures remained unchanged and stress fibers were not observed in linear networks with either Hanks balanced salt solution (HBSS) control (Fig 6I) or histamine (Fig 6J). F-actin reorganization was also studied when both coupled cells and individual cells were present in the same well by controlling the cell seeding density. Without histamine, dense F-actin structures were observed in both coupled cells and individual cells (Fig 6M). With histamine treatment, dense F-actin structures were observed only in coupled cells and cytoskeletal reorganization was observed in individual cells (Fig 6N), supporting the notion that histamine-mediated cytoskeletal reorganization depends on the cellular architecture.

To test if the effect of histamine is modulated by calcium oscillations, pulses of histamine by repetitive pipetting histamine (30 seconds) and HBSS (1 minute) were manually applied to generate calcium pulses in individual cells and monolayers (Fig 6C and 6G). The effects of histamine pulses on the actin cytoskeleton were significantly lower than continuous treatment of histamine for 5 min, despite the fact that the total treatment time were similar (Fig 6H). On the other hand, blocking calcium oscillation in cells in linear patterns via the gap junction blocker, which generate a prolonged calcium response in the network, triggered histamine-mediated cytoskeletal reorganization (Fig 6K). Dense F-actin structures were also significantly reduced by histamine for individual cells in linear cell networks (Fig 6O). There is no significant difference between histamine only and histamine with 18β-GA for individual cells (Fig 6P). These observations support the notion that cytoskeletal reorganization is triggered only by a prolonged calcium response, but not calcium oscillations.

### Collective calcium signaling regulates cell contractility

**Fig 7:**
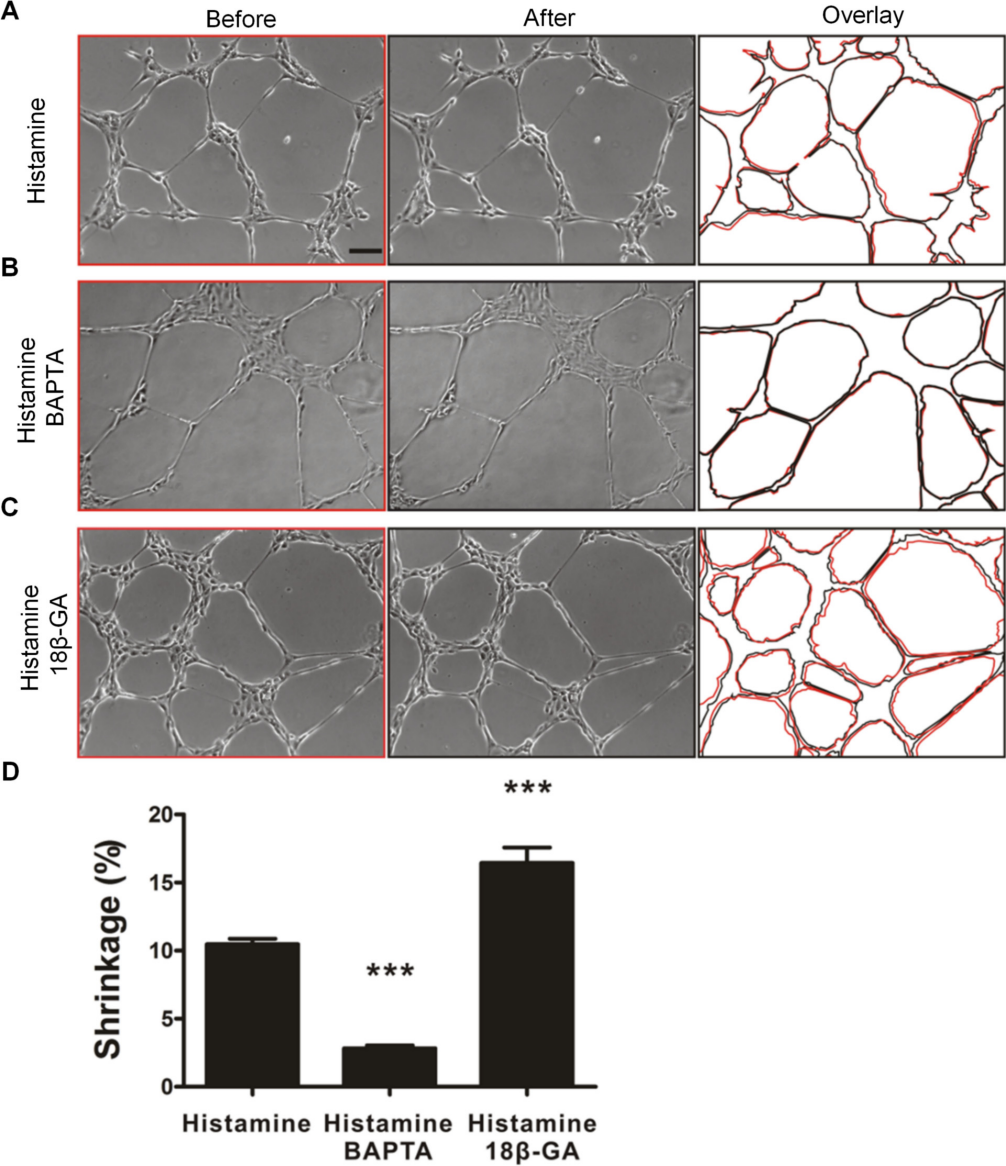
Calcium signaling regulates histamine-mediated contraction in capillary-like networks. (A-C) Shrinkage of capillary-like networks after treatment with (A) histamine, (B) histamine and BAPTA, and (C) histamine with 18β-GA. Left and middle columns indicate bright-field images of the networks before and after treatment. Right column shows overlays of cell structure profiles before (red) and after (black) histamine treatment. Scale bar, 100 μm. (D) Quantification of the relative shrinkage area. Data are representative from seven independent experiments (Bonferroni's multiple comparison test; ***, P<0.001).

Histamine-mediated cell contraction was investigated in capillary-like networks to further explore the effects and potential physiological functions of collective calcium signaling. With histamine treatment, cells contracted and migrated towards the nodes, resulting in a moderate (~10%) shrinkage of the capillary-like network (Fig 7A and S4 Video). The shrinkage was estimated by the areas of the network before and after histamine treatment. For control with HBSS, no significant changes of the networks were observed (S5 Video). With 1,2-Bis(2-aminophenoxy)ethane-N,N,N’,N’-tetraacetic acid tetrakis(acetoxymethyl ester (BAPTA-AM) pretreatment, histamine-induced calcium signaling was inhibited and the network shrinkage rate reduced to ~3% (Fig 7B). In contrast, the histamine-induced shrinkage increased to ~16% for capillary-like networks treated with a gap junction blocker which generated a prolonged calcium response (Fig 7C). The nucleus displacement was also analyzed by particle image velocimetry analysis to study histamine-induced cell contraction (S5 Fig and S6 Video). The average displacement of nuclei mediated by histamine was decreased by BAPTA and increased by 18β-GA, supporting the contraction is calcium dependent and can be enhanced by a prolonged calcium response (S6 Fig). The nuclei displacement data were in good agreement with the shrinkage data (Fig 7D and S6D Fig).

## Discussion

In this study, the significance of cellular architecture in GJIC-mediated collective calcium signaling is identified. Unlike monolayers and individual cells that exhibit prolonged cytosolic calcium response, cells in capillary-like networks and hexagonal cell networks display short calcium pulses and oscillations after histamine treatment. Histamine-mediated calcium oscillations occur only when a small number of (~2–6) cells are physically connected in self-assembled and microengineered networks. Systematic investigation using linear networks and computational modeling suggests that the calcium oscillation occurrence rate negatively correlates with the number of neighboring cells. Patterning cells in linear networks with low cell density and with different widths supports that the calcium oscillation is not due to the elongated morphology of the cells or physical confinement by the patterns. The dependence on the number of coupled cells leads to the architecture-dependent calcium signaling, since most cells in the networks connect to 2–5 cells while cells in monolayers typically connect to over 8 cells. Additionally, pharmacological inhibition, computational modeling and micropatterning revealed that the calcium oscillation is mediated by GJIC. Inhibition of GJIC with a gap junction blocker suppresses the calcium oscillation. With GJIC inhibition, the calcium dynamics of cells in capillary-like networks and hexagonal cell networks resemble the response of individual cells. These results collectively suggest that cellular architecture regulates collective calcium signaling via GJIC.

A computational model is developed to explore the mechanisms of architecture-dependent calcium oscillations. The computational model captures the essence of calcium dynamics, including the transient pulses, the uniform amplitude, the concentration dependence, the irregular pattern, the occurrence rate, and the architecture dependence. The model provides insights into the mechanisms that drive the architecture-dependent calcium oscillation. In particular, the calcium fluxes are highly sensitive to the cytosolic calcium concentration. The coupling current due to electrical coupling perturbs the cytosolic calcium concentration, which generates the oscillatory activity in coupled cells [8]. The electrical coupling provides a potential mechanism on how calcium oscillations are generated in coupled cells, but not in individual cells. Incorporating electrical coupling in the model successfully creates the random distribution of calcium pulses and the irregular pattern observed in the experiment. The oscillation cannot be explained by intercellular calcium diffusion alone, as the oscillations are irregular and unsynchronized between neighboring cells. In contrast, the biochemical coupling through intercellular diffusion of calcium suppresses the oscillation by stabilizing the cytosolic calcium concentration in the cells. As supported by the computational model and linear networks with different widths, the stabilizing capability increases with the number of cells coupled and abolishes calcium oscillations when a large number of cells are connected (e.g., in monolayers). Since the stabilizing capability depends on the number of neighboring cells, the calcium oscillation occurs only when a small number of cells are coupled, providing a basis of the architecture-dependent calcium signaling (S7 and S8 Figs).

Our computational model is based on a set of assumptions and simplifications, which will likely limit the predictive power of the model. Several aspects of the model should be considered. First, the contribution of voltage gated calcium channels to agonist-induced calcium entry in endothelial cells is inconclusive [33–35]. Endothelial cells are often considered to be electrically unexcitable and the effect of membrane potential on agonist-induced calcium signaling in endothelial cells remains controversial [36, 37]. Second, the permeability of vertebrate gap junction can be modulated by cytosolic ionic composition and connexin phosphorylation. The increase in cytosolic calcium concentration was shown to inhibit electrical coupling and dye transfer among cells [38]. Phosphorylation of connexin by protein kinases also resulted in internalization and degradation of gap junctions [39]. Our model may only represent the cell behaviors under specific conditions and do not rule out the possibility of other calcium signaling mechanisms.

In general, calcium dynamics can be influenced by a large number of factors and mechanisms. Computational models of calcium dynamics and signaling in vascular regulation have been reviewed previously [40]. The relationships between the cytosolic calcium, membrane potential, coupling current and voltage gated channels can be context-dependent (e.g., agonist, concentration and duration) and each factor may influence the calcium dynamics through multiple feedback loops direct or indirectly. As an example, the inhibitory effects of verapamil (an L-type voltage gated calcium channels inhibitor) on histamine-induced calcium signaling in human umbilical vein endothelial cells were observed only at submaximal histamine concentrations, suggesting that voltage gated calcium channels might play a role at a specific range of agonist concentration in HUVECs [41]. Non-selective cation channels were also reported to be responsible for histamine-activated Ca^2+^ influx [42]. For histamine-activated calcium signaling in endothelial cells, it has been demonstrated that the membrane potential plays a key role for fine-tuning the calcium influx [43]. In particular, the membrane potential can effectively regulate calcium influx which is responsible for the plateau phase of agonist-mediated calcium dynamics. In addition, activation of the calcium-activated potassium channel triggers membrane hyperpolarization and, thus, provides a driving force for cation influx (Na^+^, K^+^ and Ca^2+^) [44]. In the future, additional studies should be performed to determine the contribution of voltage-gated calcium channels, membrane potential and gap junction mediated electrical coupling with the regulation of cytosolic calcium concentration and connexin phosphorylation.

Our results underscore the possibility that cellular architecture modulates histamine-mediated cytoskeletal reorganization and cell contractility via GJIC-mediated collective calcium signaling. Unlike monolayers and individual cells that exhibit cytoskeletal reorganization, histamine has minimal effects on the actin cytoskeleton in capillary-like networks and hexagonal cell networks. To study the roles of calcium oscillation on cytoskeletal reorganization, histamine pulses that impose calcium oscillations are applied to individual cells and monolayers. The histamine pulses have a weak effect on the actin cytoskeleton compared to continuous histamine treatment. On the other hand, suppressing calcium oscillations in self-assembled networks by GJIC inhibition induces cytoskeletal reorganization and cell contraction. Therefore, the effects of histamine can be modulated by the calcium dynamics and are architecture-dependent. Physiologically, the architecture dependence may represent one of the ways of endothelial cells to respond to the stimuli differentially in various vascular structures. The architecture-dependent calcium oscillations might contribute to the specificity of histamine-induced permeability in venules, but not in capillaries [15]. Our results, however, do not rule out other potential mechanisms in generating the specificity such as the differences in calcium signaling mechanisms between venules and capillaries. Further investigation is required to test this hypothesis and study the architecture dependence in other models (S9 Fig). As a universal second messenger, collective calcium signaling may serve as a broad mechanism in regulating various cell functions. Other factors may also be considered in the computational and experimental models to decipher the regulation and functional implication of collective calcium signaling in diverse cellular architectures.

## Materials and Methods

### Cell culture

HUVEC were purchased from BD Bioscience (Bedford, MA). The cells were cultured in Medium 200 with low serum growth supplement (Life Technologies, Carlsbad, CA) and 5% CO_2_ at 37°C. HUVEC from passages three to six were used in all experiments.

### Capillary-like network formation

Matrigel (growth factor reduced, BD Bioscience) was thawed with ice at 4°C refrigerator overnight, added into 48-well plates (130 μL per well) and gelled at 37°C for 1 hour. HUVEC were harvested from tissue culture dishes at a confluency of ~90% using 0.25% Trypsin-EDTA (Life Technologies) for 1−2 minutes). The cells were then seeded on top of the gelled matrigel at a density of 300−400 cells/mm^2^. At 8 hours after cell seeding, images of capillary-like networks were taken by a CCD camera (Cooke SensiCam) with an epi-fluorescence microscope (Nikon TE2000-U).

### Cell patterning

Plasma lithography was employed to pattern cells into hexagonal cell networks that mimic the capillary, and into linear cell networks for studying the effects of the number of neighboring cells on collective calcium signaling. Briefly, a positive master (50 μm in height) on silicon wafer was fabricated with SU-8 (MicroChem) by photolithography. The polydimethylsiloxane (PDMS) pre-polymers (Sylgard 184, Dow Corning) were casted on the master and cured at 65°C for 4 hours. The widths of linear patterns were 20 μm, 40 μm and 100 μm. The width and segment length of hexagonal channels were 20 μm and 250 μm respectively. The mold was placed in conformal contact on a native polystyrene substrate (24-well plate from BD Bioscience), exposed to atmospheric plasma for 20 minutes and removed. The HUVEC were then seeded (800 cells/mm^2^) onto the substrate. After overnight culture, cells selectively adhered to the plasma treated area and formed microengineered cell networks.

### Calcium imaging and analysis

Cell networks were washed gently with HBSS for three times, incubated with 10 μM Fluo-3/AM (Biotium) in HBSS at 37°C for 45 minutes in the dark, and washed with HBSS for another three times to remove the residual Fluo-3/AM. Fluorescence images were captured at 100 ms intervals with an exposure time of 400 ms for 5 minutes once the histamine (Sigma) solution was applied to the cells with a pipette at a final concentration of 5 μM. Since the largest frequency of calcium oscillations found in non-excited cells is about 0.1 Hz, 400 ms exposure time should capture all the calcium spikes [45]. The images were analyzed by ImageJ (NIH). To investigate the effect of gap junctions on collective calcium signaling, cells were pre-incubated with 40 μM 18β-GA (Sigma) for 30 min and stimulated with histamine in the presence of 18β-GA. The relative change in calcium was calculated as previously mentioned [46]. The frequency of calcium oscillations was estimated by utilizing the time interval between the first two calcium spikes. To quantitatively differentiate calcium oscillations that have two or more calcium spikes from small and short repetitive fluctuations in cytosolic calcium, amplitude of the calcium signal relative to the baseline noise was used. A calcium fluctuation with peak-to-baseline ratio larger than 3 was counted as a calcium spike.

### Immunostaining and imaging

Cells were fixed with 4% paraformaldehyde for 15 minutes, washed with PBS twice, permeabilized with 0.2% (w/v) Triton X-100 (Sigma) for at least one hour, washed with PBS twice and blocked with 1% BSA (sigma) for 5 hours. Alexa Fluor 555 Phalloidin (Life Technologies) was diluted at 1:30 ratio with 1% BSA solutions and applied to the fixed cells at 4°C overnight. Cells were washed three times with PBS (5 minutes each time) before imaging. For histamine H1 receptor immunofluorescence, cells were fixed and blocked as described above, and incubated overnight at 4°C with rabbit histamine H1 receptor antibody (Novus Biologicals, rabbit polyclonal IgG, 1:150 diluted). After washing three times with PBS, cells were further incubated at room temperature for 1 hour with Alexa Fluor® 555 donkey anti-rabbit IgG antibody (Life Technologies). To study the roles of calcium signaling in cytoskeletal reorganization, cells were incubated with 15 μM BAPTA-AM (EMD Millipore) for 30 minutes at 37°C in the dark and washed three times with HBSS before histamine applications. To further resolve the role of calcium oscillations, three pulses (30 seconds) of histamine solution were applied to the monolayer and single cells with an interval of 1 minute HBSS wash between pulses to mimic natural calcium oscillations.

### Capillary-like network shrinkage and particle image velocimetry (PIV) analysis

For estimating the shrinkage ratio of capillary-like networks, ImageJ was used for measuring the areas of cell networks before drug application and 5 minutes after drug application. For PIV analysis, cell nuclei were stained with Hoechst 33342 (Life Technologies) for 20 minutes at 37°C, and fluorescence images (blue) of capillary-like networks before and after drug applications were analyzed with ImageJ and PIV plugins.

### Statistical analysis

Student’s unpaired t-tests (two-tailed) were performed to compare two experimental groups. A one-way ANOVA with Bonferroni post-tests was used to compare the means of three or more experimental groups. All data are presented as mean ± standard error of the mean (SEM).

## Supporting Information

S1 Fig Plasma lithography cell patterning creates microengineered networks of endothelial cells. (A) A PDMS template with linear or hexagonal patterns is fabricated by photolithography and PDMS molding. The PMDS template is placed in a polystyrene well with a weight to create conformal contact between the template and the substrate. (B) The PDMS-shielded polystyrene well is exposed to atmospheric plasma for 20 minutes to selectively functionalize the polystyrene surface. (C) Cells are seeded in the well and selectively adhere to the plasma-treated regions to create the microengineered networks. (D) A bright-field image of linear cell networks. Scale bar, 50 μm. (E-F) Bright-field images of capillary-like and hexagonal cell networks formed by HUVEC. Scale bar, 200 μm. (G-H) Fluorescence images of cell nuclei stained with Hoechst 33342 in capillary-like and hexagonal cell networks. Images are representative from three independent experiments.
(TIF)

S2 Fig Gap junction inhibition abolishes calcium oscillations in capillary-like and hexagonal cell networks. (A-B) Histamine-induced calcium signaling in (A) capillary-like networks and (B) hexagonal cell networks with the presence of a gap junction blocker, 18β-GA. The calcium response curves were shifted vertically for clarity. Red arrows indicate the time of histamine addition. Data are representative from three independent experiments.
(TIF)

S3 Fig Computational simulation of two coupled cells. (A) The electrical coupling strengths (*g*_*ij*_) for uncoupled and coupled cells were 0 and 1000 μS/cm^2^ respectively. (B) Influences of electrical (*g*_*ij*_) and biochemical (*D*_*ij*_) coupling strengths on the calcium dynamics of two coupled cells. The data were shifted vertically for clarity.
(TIF)

S4 Fig Percentage of cells at the edge (not surrounded on all sides) and in the inner region (surrounded on all sides) that underwent calcium oscillations (n = 3 for capillary-like networks (CLN); n = 8 for hexagonal cell networks (Hexagon) and 100 μm linear cell networks; **, P<0.01; ***, P<0.001). (TIF)

S5 Fig **Nuclei displacements (μm) of cell in capillary-like structures measured by particle image velocimetry at the presence of (A) histamine only, (B) histamine with BAPTA, and (C) histamine with 18β-GA.** Data are representative from five independent experiments.
(TIF)

S6 Fig Cell contraction and remodeling of capillary-like networks. (A–C) Nuclei displacement before and after cells were treated with (A) histamine only, (B) histamine and BAPTA, (C) histamine and 18β-GA. Scale bar, 100 μm. (D) Statistical analysis of mean nuclei displacement in cells in capillary-like networks with histamine only, histamine with BAPTA and histamine with 18β-GA (Bonferroni's multiple comparison test; n = 5; **, P<0.01; ***, P<0.001).
(TIF)

S7 Fig Computational modeling of architecture-dependent calcium signaling. Effects of the number of coupled cells on cytosolic calcium, ER calcium, and membrane potential in 3, 4, and 6 cells connected. The data were shifted vertically for clarity.
(TIF)

S8 Fig Computational modeling of architecture-dependent calcium signaling. Effects of the calcium diffusivity of coupled cells on cytosolic calcium, ER calcium, and membrane potential. The data were shifted vertically for clarity.
(TIF)

S9 Fig Distributions of histamine H1 receptor in different cell structures. (A-D) Immunofluorescence staining of histamine H1 receptors (red) and nuclei (blue) in (A) monolayers, (B) linear cell networks, (C) capillary-like networks, and (D) hexagonal cell networks. Scale bar, 40 μm.
(TIF)

S1 Video Calcium signaling of individual cells. The cell density was approximately 100 cells/mm^2^. The cells were treated with 5 μM histamine. The field of view of the movie was 860 by 688 μm. Considering the video size, fluorescence images taken every 1 second (half of original acquired images) were used. The duration of the video is 250 seconds.
(MOV)

S2 Video Calcium signaling of monolayers. The cell density was approximately 800 cells/mm^2^. The cells were treated with 5 μM histamine. The field of view was 860 by 688 μm. Fluorescence images were acquired at 1 frame/s. The duration of the video is 250 seconds.
(MOV)

S3 Video Calcium signaling in capillary-like networks formed by HUVEC on matrigel. The cells were treated with 5 μM histamine. The field of view was 860 by 688 μm. Fluorescence images were acquired at 1 frame/second. The duration of the video is 250 seconds.
(MOV)

S4 Video Histamine induced shrinkage of a capillary-like network. The cells were treated with 5 μM histamine. The field of view was 860 by 688 μm. Fluorescence images were acquired at 5 frame/second. The duration of the video is 250 seconds.
(MOV)

S5 Video Capillary-like network were treated with HBSS control. The field of view was 860 by 688 μm. Fluorescence images were acquired at 5 frame/second. The duration of the video is 250 seconds.
(MOV)

S6 Video Nuclei displacements of cells in a capillary-like network. The cells were treated with 5 μM histamine. The field of view was 935 by 698 μm. Fluorescence images were acquired at 5 frame/second. The duration of the video is 250 seconds.
(MOV)

S1 Text Supplementary note. Computational modeling of pluricellular calcium dynamics.
(DOCX)

## References

[1] Berridge MJ, Bootman MD, Roderick HL. Calcium signalling: Dynamics, homeostasis and remodelling. Nat Rev Mol Cell Bio. 2003;4(7):517–29. 12838335 10.1038/nrm1155

[2] Berridge MJ, Lipp P, Bootman MD. The versatility and universality of calcium signalling. Nature reviews Molecular cell biology. 2000;1(1):11–21. Epub 2001/06/20. 11413485 10.1038/35036035

[3] Clapham DE. Calcium signaling. Cell. 2007;131(6):1047–58. 18083096 10.1016/j.cell.2007.11.028

[4] Dolmetsch RE, Xu KL, Lewis RS. Calcium oscillations increase the efficiency and specificity of gene expression. Nature. 1998;392(6679):933–6. 9582075 10.1038/31960

[5] Parekh AB. Decoding cytosolic Ca2+ oscillations. Trends Biochem Sci. 2011;36(2):78–87. 10.1016/j.tibs.2010.07.013 20810284

[6] Kupzig S, Walker SA, Cullen PJ. The frequencies of calcium oscillations are optimized for efficient calcium-mediated activation of Ras and the ERK/MAPK cascade. P Natl Acad Sci USA. 2005;102(21):7577–82. 10.1073/pnas.0409611102 PMC1103707 15890781

[7] Harris AL, Spray DC, Bennett MVL. Control of intercellular communication by voltage dependence of gap junctional conductance. J Neurosci. 1983;3(1):79–100. 6822860 10.1523/JNEUROSCI.03-01-00079.1983 PMC6564587

[8] Loewenstein Y, Yarom Y, Sompolinsky H. The generation of oscillations in networks of electrically coupled cells. Proc Natl Acad Sci USA. 2001;98(14):8095–100. 11427705 10.1073/pnas.131116898 PMC35473

[9] Demer LL, Wortham CM, Dirksen ER, Sanderson MJ. Mechanical stimulation induces intercellular calcium signaling in bovine aortic endothelial-cells. Am J Physiol. 1993;264(6):H2094–H102. 8322938 10.1152/ajpheart.1993.264.6.H2094

[10] Junkin M, Lu Y, Long JX, Deymier PA, Hoying JB, Wong PK. Mechanically induced intercellular calcium communication in confined endothelial structures. Biomaterials. 2013;34(8):2049–56. 10.1016/j.biomaterials.2012.11.060 23267827 PMC3542404

[11] Hraha TH, Bernard AB, Nguyen LM, Anseth KS, Benninger RK. Dimensionality and size scaling of coordinated Ca(2+) dynamics in MIN6 beta-cell clusters. Biophysical Journal. 2014;106(1):299–309. Epub 2014/01/15. 10.1016/j.bpj.2013.11.026 24411262 PMC3907241

[12] Schumacher JA, Hsieh YW, Chen SH, Pirri JK, Alkema MJ, Li WH, et al Intercellular calcium signaling in a gap junction-coupled cell network establishes asymmetric neuronal fates in C. elegans. Development. 2012;139(22):4191–201. 10.1242/dev.083428 23093425 PMC3478688

[13] Sun B, Lembong J, Normand V, Rogers M, Stone HA. Spatial-temporal dynamics of collective chemosensing. Proc Natl Acad Sci USA. 2012;109(20):7753–8. 10.1073/pnas.1121338109 22566661 PMC3356672

[14] Kameritsch P, Pogoda K, Ritter A, Munzing S, Pohl U. Gap junctional communication controls the overall endothelial calcium response to vasoactive agonists. Cardiovascular Research. 2012;93(3):508–15. Epub 2011/12/27. 10.1093/cvr/cvr345 22198510

[15] Michel CC, Curry FE. Microvascular permeability. Physiological Reviews. 1999;79(3):703–61. 10390517 10.1152/physrev.1999.79.3.703

[16] Sun J, Jamilpour N, Wang FY, Wong PK. Geometric control of capillary architecture via cell-matrix mechanical interactions. Biomaterials. 2014;35(10):3273–80. Epub 2014/01/21. 10.1016/j.biomaterials.2013.12.101 24439400 PMC3925070

[17] Long J, Junkin M, Wong PK, Hoying J, Deymier P. Calcium Wave Propagation in Networks of Endothelial Cells: Model-based Theoretical and Experimental Study. Plos Comput Biol. 2012;8(12):e1002847 Epub 2013/01/10. 10.1371/journal.pcbi.1002847 23300426 PMC3531288

[18] Junkin M, Watson J, Geest JPV, Wong PK. Template-guided self-assembly of colloidal quantum dots using plasma lithography. Adv Mater. 2009;21(12):1247–51.

[19] Junkin M, Wong PK. Probing cell migration in confined environments by plasma lithography. Biomaterials. 2011;32(7):1848–55. 10.1016/j.biomaterials.2010.11.009 21134692 PMC3023939

[20] Junkin M, Leung SL, Yang Y, Lu Y, Volmering J, Wong PK. Plasma lithography surface patterning for creation of cell networks. J Vis Exp. 2011;52. 10.3791/3115 PMC3197071 21694697

[21] Junkin M, Leung SL, Whitman S, Gregorio CC, Wong PK. Cellular self-organization by autocatalytic alignment feedback. Journal of cell science. 2011;124(24):4213–20. 22193956 10.1242/jcs.088898 PMC3258106

[22] Yang Y, Jamilpour N, Yao B, Dean ZS, Riahi R, Wong PK. Probing Leader Cells in Endothelial Collective Migration by Plasma Lithography Geometric Confinement. Scientific reports. 2016;6:22707 Epub 2016/03/05. 10.1038/srep22707 26936382 PMC4776176

[23] Jacob R, Merritt JE, Hallam TJ, Rink TJ. Repetitive spikes in cytoplasmic calcium evoked by histamine in human endothelial cells. Nature. 1988;335(6185):40–5. Epub 1988/09/01. 3412458 10.1038/335040a0

[24] Laird DW. The gap junction proteome and its relationship to disease. Trends Cell Biol. 2010;20(2):92–101. Epub 2009/12/01. 10.1016/j.tcb.2009.11.001 19944606

[25] Levin M. Gap junctional communication in morphogenesis. Progress in Biophysics & Molecular Biology. 2007;94(1–2):186–206. 10.1016/j.pbiomolbio.2007.03.005 PMC2292839 17481700

[26] Yamamoto Y, Fukutiz H, Nakahira Y, Suzuki H. Blockade by 18 beta-glycyrrhetinic acid of intercellular electrical coupling in guinea-pig arterioles. J Physiol-London. 1998;511(2):501–8. 9706026 10.1111/j.1469-7793.1998.501bh.x PMC2231143

[27] Dey A, Kusljic S, Lang RJ, Exintaris B. Role of connexin 43 in the maintenance of spontaneous activity in the guinea pig prostate gland. Brit J Pharmacol. 2010;161(8):1692–707. 20735413 10.1111/j.1476-5381.2010.01001.x PMC3010576

[28] Sneyd J, Tsaneva-Atanasova K, Yule DI, Thompson JL, Shuttleworth TJ. Control of calcium oscillations by membrane fluxes. Proc Natl Acad Sci U S A. 2004;101(5):1392–6. Epub 2004/01/22. 14734814 10.1073/pnas.0303472101 PMC337063

[29] Majno G, Shea SM, Leventhal M. Endothelial contraction induced by histamine-type mediators: an electron microscopic study. The Journal of cell biology. 1969;42(3):647–72. Epub 1969/09/01. 5801425 10.1083/jcb.42.3.647 PMC2107712

[30] Wu NZ, Baldwin AL. Transient Venular Permeability Increase and Endothelial Gap Formation Induced by Histamine. American Journal of Physiology. 1992;262(4):H1238–H47. 1566906 10.1152/ajpheart.1992.262.4.H1238

[31] Ehringer WD, Edwards MJ, Miller FN. Mechanisms of alpha-thrombin, histamine, and bradykinin induced endothelial permeability. Journal of Cellular Physiology. 1996;167(3):562–9. 8655610 10.1002/(SICI)1097-4652(199606)167:3<562::AID-JCP20>3.0.CO;2-4

[32] van Nieuw Amerongen GP, Draijer R, Vermeer MA, van Hinsbergh VW. Transient and prolonged increase in endothelial permeability induced by histamine and thrombin: role of protein kinases, calcium, and RhoA. Circ Res. 1998;83(11):1115–23. Epub 1998/12/01. 9831706 10.1161/01.res.83.11.1115

[33] Welsh DG, Tran CH, Plane F, Sandow S. Letter to the editor: "Are voltage-dependent ion channels involved in the endothelial cell control of vasomotor tone?". Am J Physiol Heart Circ Physiol. 2007;293(3):H2007; author reply H8. Epub 2007/09/07. 17804398 10.1152/ajpheart.00722.2007

[34] Figueroa XF, Chen CC, Campbell KP, Damon DN, Day KH, Ramos S, et al Are voltage-dependent ion channels involved in the endothelial cell control of vasomotor tone? Am J Physiol Heart Circ Physiol. 2007;293(3):H1371–83. Epub 2007/05/22. 17513486 10.1152/ajpheart.01368.2006

[35] Tran QK, Ohashi K, Watanabe H. Calcium signalling in endothelial cells. Cardiovasc Res. 2000;48(1):13–22. Epub 2000/10/18. 11033104 10.1016/s0008-6363(00)00172-3

[36] Cohen KD, Jackson WF. Membrane hyperpolarization is not required for sustained muscarinic agonist-induced increases in intracellular Ca2+ in arteriolar endothelial cells. Microcirculation. 2005;12(2):169–82. Epub 2005/04/13. 15824039 10.1080/10739680590904973 PMC1405751

[37] McSherry IN, Spitaler MM, Takano H, Dora KA. Endothelial cell Ca2+ increases are independent of membrane potential in pressurized rat mesenteric arteries. Cell Calcium. 2005;38(1):23–33. Epub 2005/05/24. 15907999 10.1016/j.ceca.2005.03.007

[38] Peracchia C. Chemical gating of gap junction channels; roles of calcium, pH and calmodulin. Biochimica et biophysica acta. 2004;1662(1–2):61–80. Epub 2004/03/23. 15033579 10.1016/j.bbamem.2003.10.020

[39] Lampe PD, Lau AF. The effects of connexin phosphorylation on gap junctional communication. The international journal of biochemistry & cell biology. 2004;36(7):1171–86. Epub 2004/04/28. 15109565 10.1016/S1357-2725(03)00264-4 PMC2878204

[40] Tsoukias NM. Calcium dynamics and signaling in vascular regulation: computational models. Wiley Interdiscip Rev Syst Biol Med. 2011;3(1):93–106. Epub 2010/11/10. 10.1002/wsbm.97 21061306 PMC3681511

[41] Rotrosen D, Gallin JI. Histamine type I receptor occupancy increases endothelial cytosolic calcium, reduces F-actin, and promotes albumin diffusion across cultured endothelial monolayers. The Journal of cell biology. 1986;103(6 Pt 1):2379–87. Epub 1986/12/01. 3782301 10.1083/jcb.103.6.2379 PMC2114605

[42] Nilius B, Schwartz G, Oike M, Droogmans G. Histamine-activated, non-selective cation currents and Ca2+ transients in endothelial cells from human umbilical vein. Pflugers Arch. 1993;424(3–4):285–93. Epub 1993/08/01. 7692391 10.1007/BF00384354

[43] Kamouchi M, Droogmans G, Nilius B. Membrane potential as a modulator of the free intracellular Ca2+ concentration in agonist-activated endothelial cells. Gen Physiol Biophys. 1999;18(2):199–208. 10517293

[44] Paltauf-Doburzynska J, Frieden M, Spitaler M, Graier WF. Histamine-induced Ca2+ oscillations in a human endothelial cell line depend on transmembrane ion flux, ryanodine receptors and endoplasmic reticulum Ca2+-ATPase. J Physiol-London. 2000;524(3):701–13. 10790152 10.1111/j.1469-7793.2000.00701.x PMC2269898

[45] Boulware MJ, Marchant JS. Timing in cellular Ca2+ signaling. Curr Biol. 2008;18(17):R769–R76. Epub 2008/09/13. 10.1016/j.cub.2008.07.018S0960-9822(08)00884-1 [pii]. 18786382 PMC3236564

[46] Sun J, Chen P, Feng XJ, Du W, Liu BF. Development of a microfluidic cell-based biosensor integrating a millisecond chemical pulse generator. Biosensors & Bioelectronics. 2011;26(8):3413–9. 21334189 10.1016/j.bios.2011.01.013

