# Supplementary figures and images for: Cellular Architecture Regulates Collective Calcium Signaling and Cell Contractility
Source: PLoS Comput Biol. 2016 May 19;12(5):e1004955. doi: 10.1371/journal.pcbi.1004955 (PMC4873241; doi:10.1371/journal.pcbi.1004955)

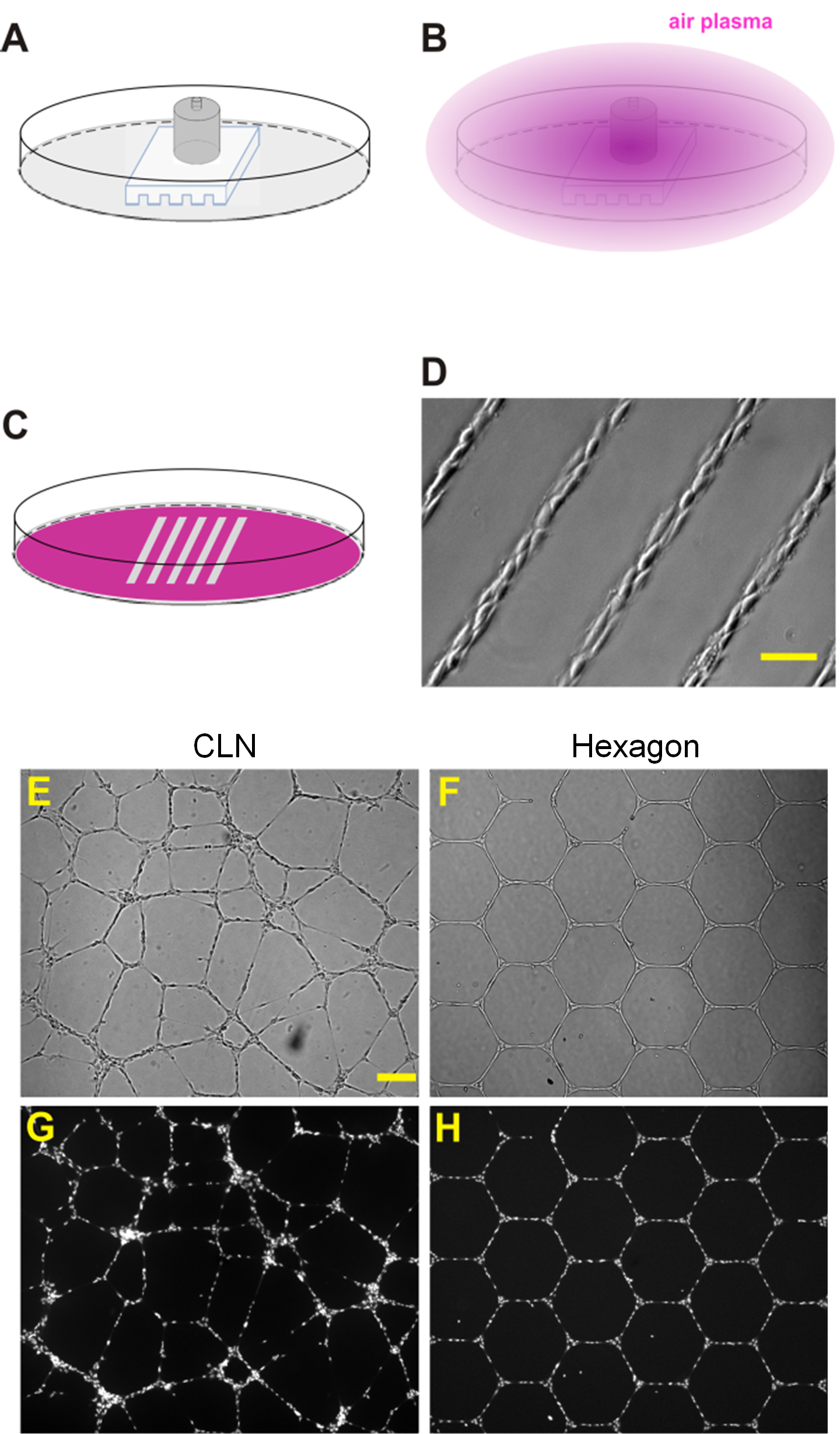

Supplement: S1 Fig — (A) A PDMS template with linear or hexagonal patterns is fabricated by photolithography and PDMS molding. The PMDS template is placed in a polystyrene well with a weight to create conformal contact between the template and the substrate. (B) The PDMS-shielded polystyrene well is exposed to atmospheric plasma for 20 minutes to selectively functionalize the polystyrene surface. (C) Cells are seeded in the well and selectively adhere to the plasma-treated regions to create the microengineered networks. (D) A bright-field image of linear cell networks. Scale bar, 50 μm. (E-F) Bright-field images of capillary-like and hexagonal cell networks formed by HUVEC. Scale bar, 200 μm. (G-H) Fluorescence images of cell nuclei stained with Hoechst 33342 in capillary-like and hexagonal cell networks. Images are representative from three independent experiments. (TIF) [file pcbi.1004955.s001.tif]

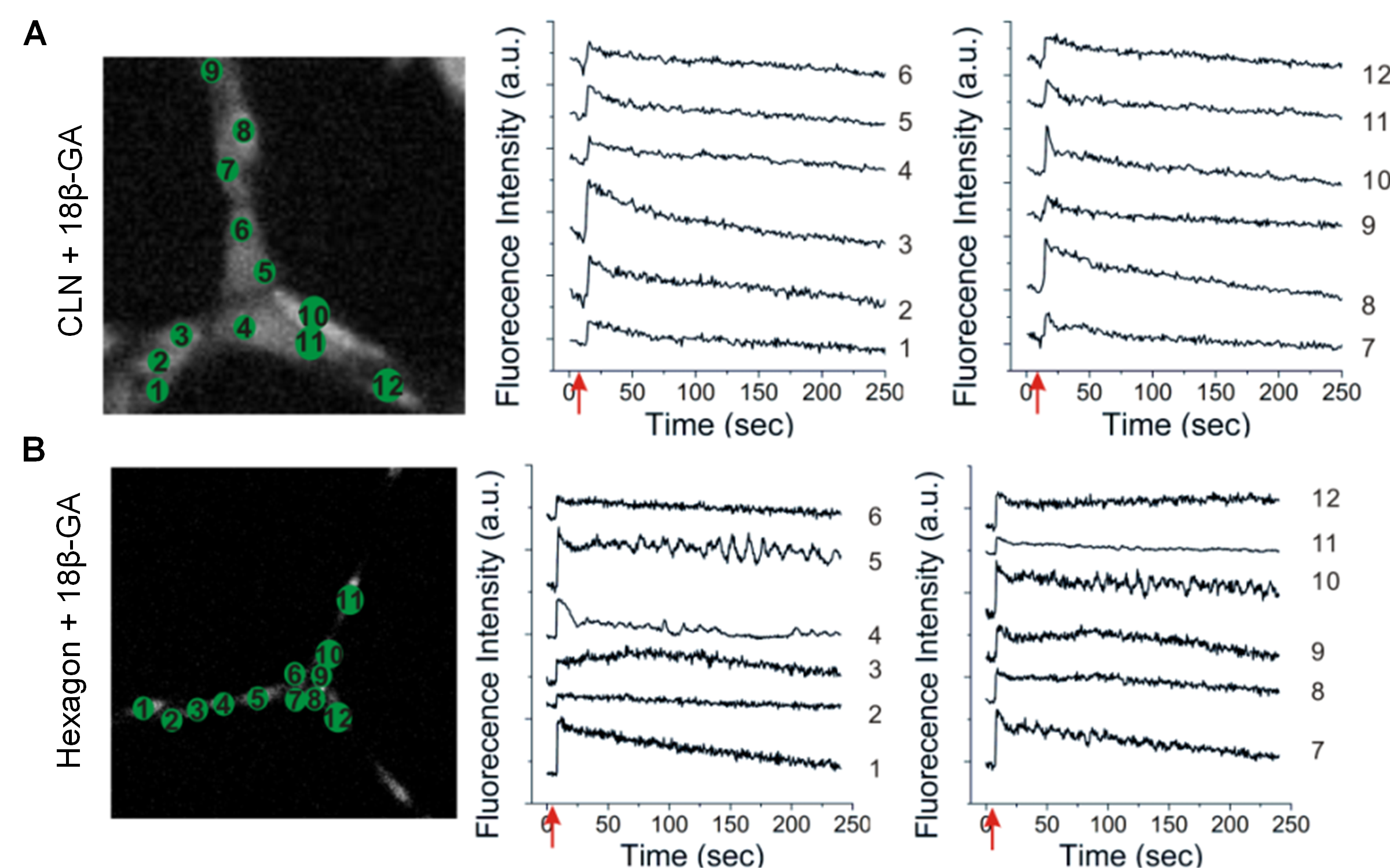

Supplement: S2 Fig — (A-B) Histamine-induced calcium signaling in (A) capillary-like networks and (B) hexagonal cell networks with the presence of a gap junction blocker, 18β-GA. The calcium response curves were shifted vertically for clarity. Red arrows indicate the time of histamine addition. Data are representative from three independent experiments. (TIF) [file pcbi.1004955.s002.tif]

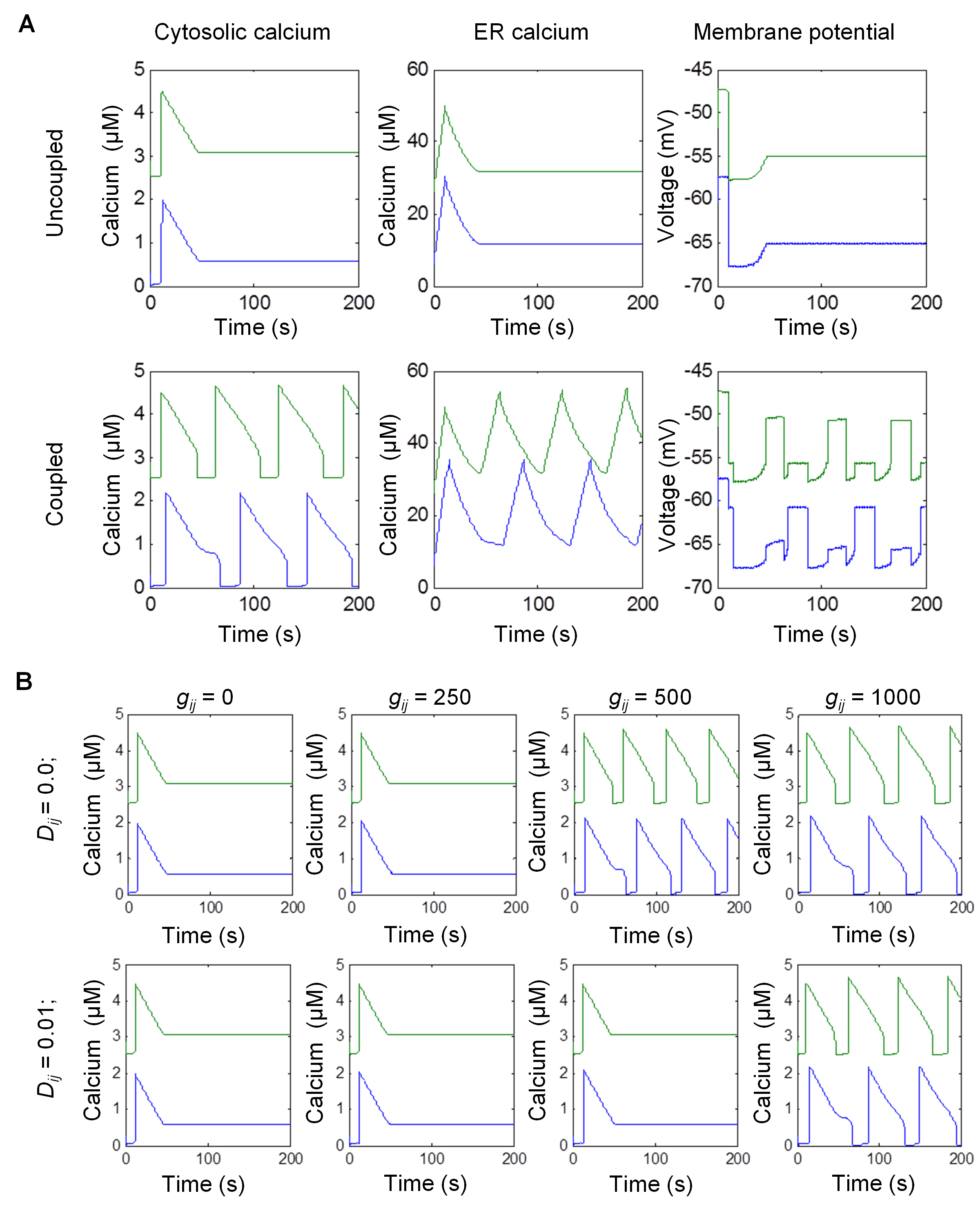

Supplement: S3 Fig — (A) The electrical coupling strengths (gij) for uncoupled and coupled cells were 0 and 1000 μS/cm2 respectively. (B) Influences of electrical (gij) and biochemical (Dij) coupling strengths on the calcium dynamics of two coupled cells. The data were shifted vertically for clarity. (TIF) [file pcbi.1004955.s003.tif]

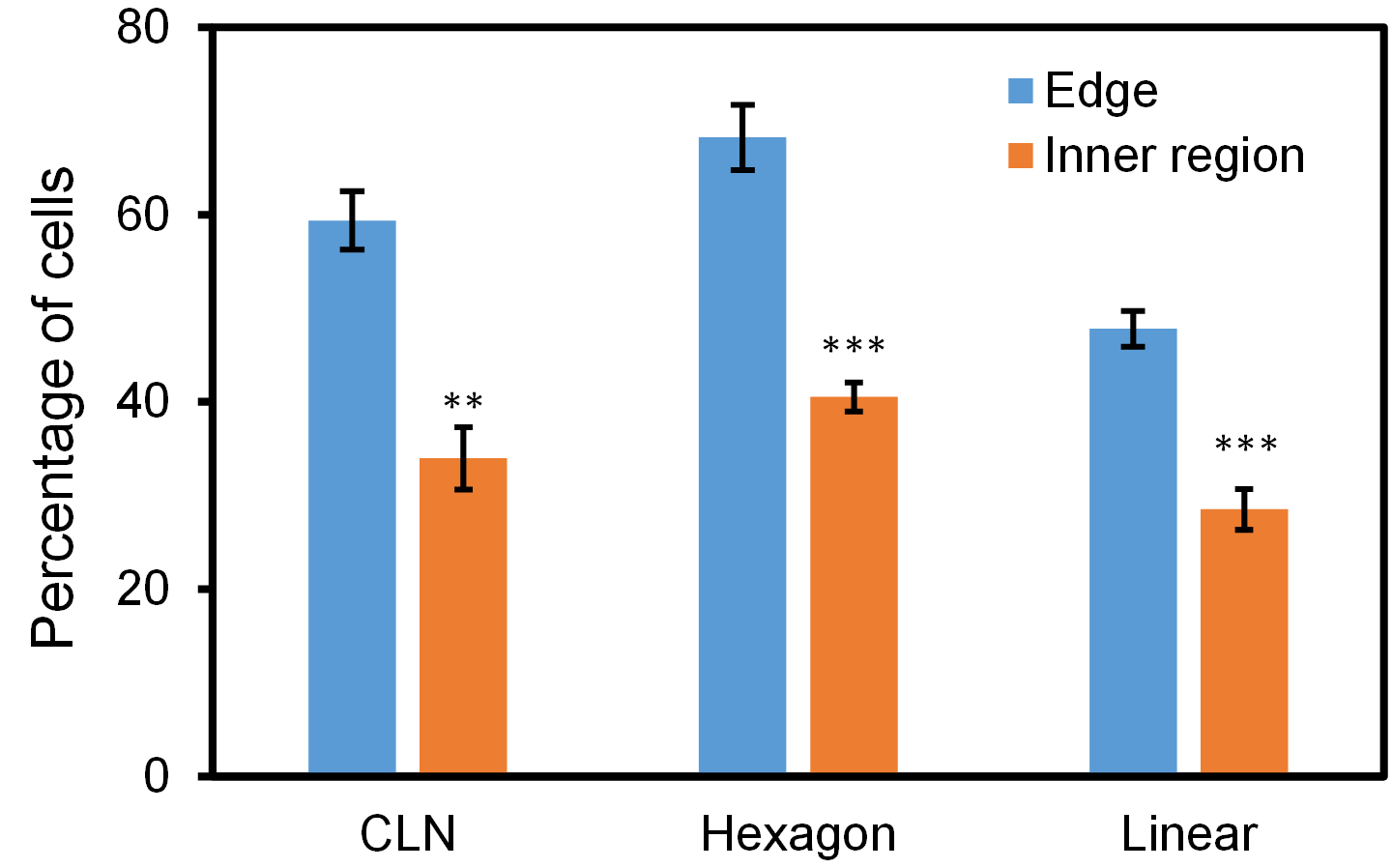

Supplement: S4 Fig — (TIF) [file pcbi.1004955.s004.tif]

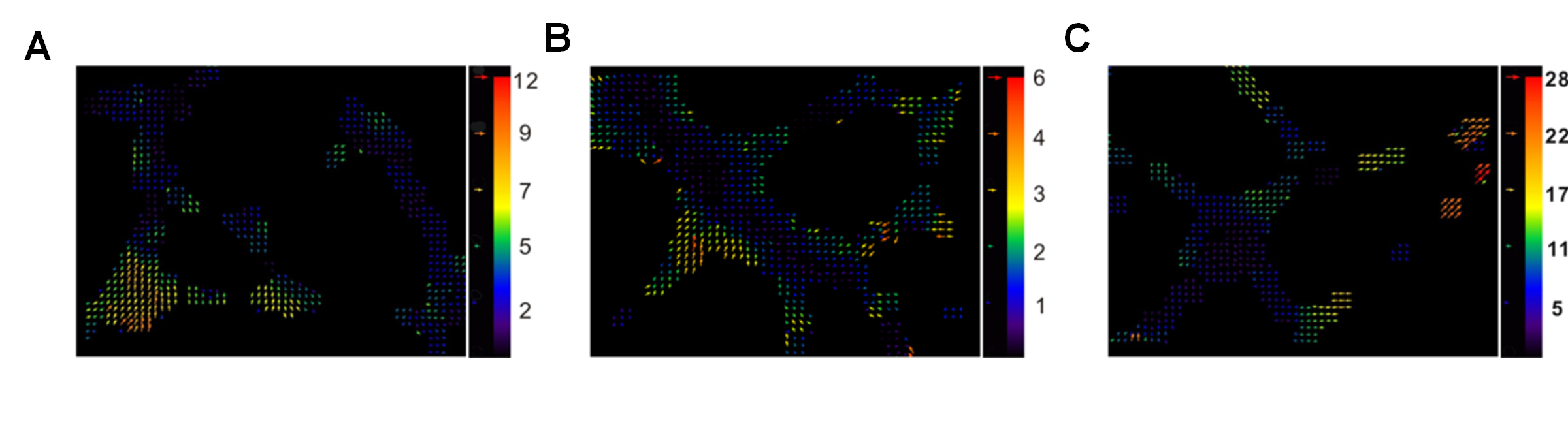

Supplement: S5 Fig — Nuclei displacements (μm) of cell in capillary-like structures measured by particle image velocimetry at the presence of (A) histamine only, (B) histamine with BAPTA, and (C) histamine with 18β-GA. Data are representative from five independent experiments. (TIF) [file pcbi.1004955.s005.tif]

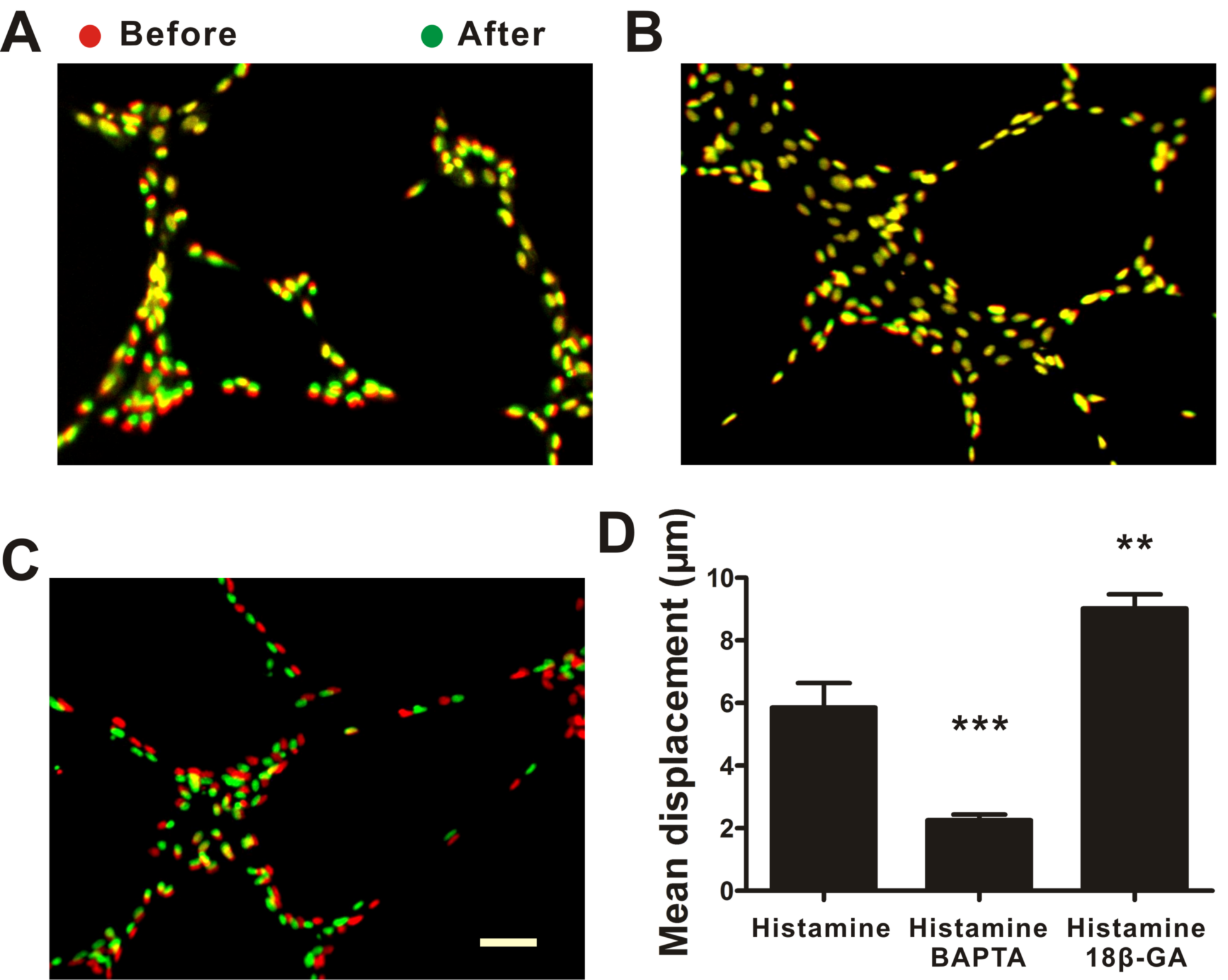

Supplement: S6 Fig — (A–C) Nuclei displacement before and after cells were treated with (A) histamine only, (B) histamine and BAPTA, (C) histamine and 18β-GA. Scale bar, 100 μm. (D) Statistical analysis of mean nuclei displacement in cells in capillary-like networks with histamine only, histamine with BAPTA and histamine with 18β-GA (Bonferroni's multiple comparison test; n = 5; **, P<0.01; ***, P<0.001). (TIF) [file pcbi.1004955.s006.tif]

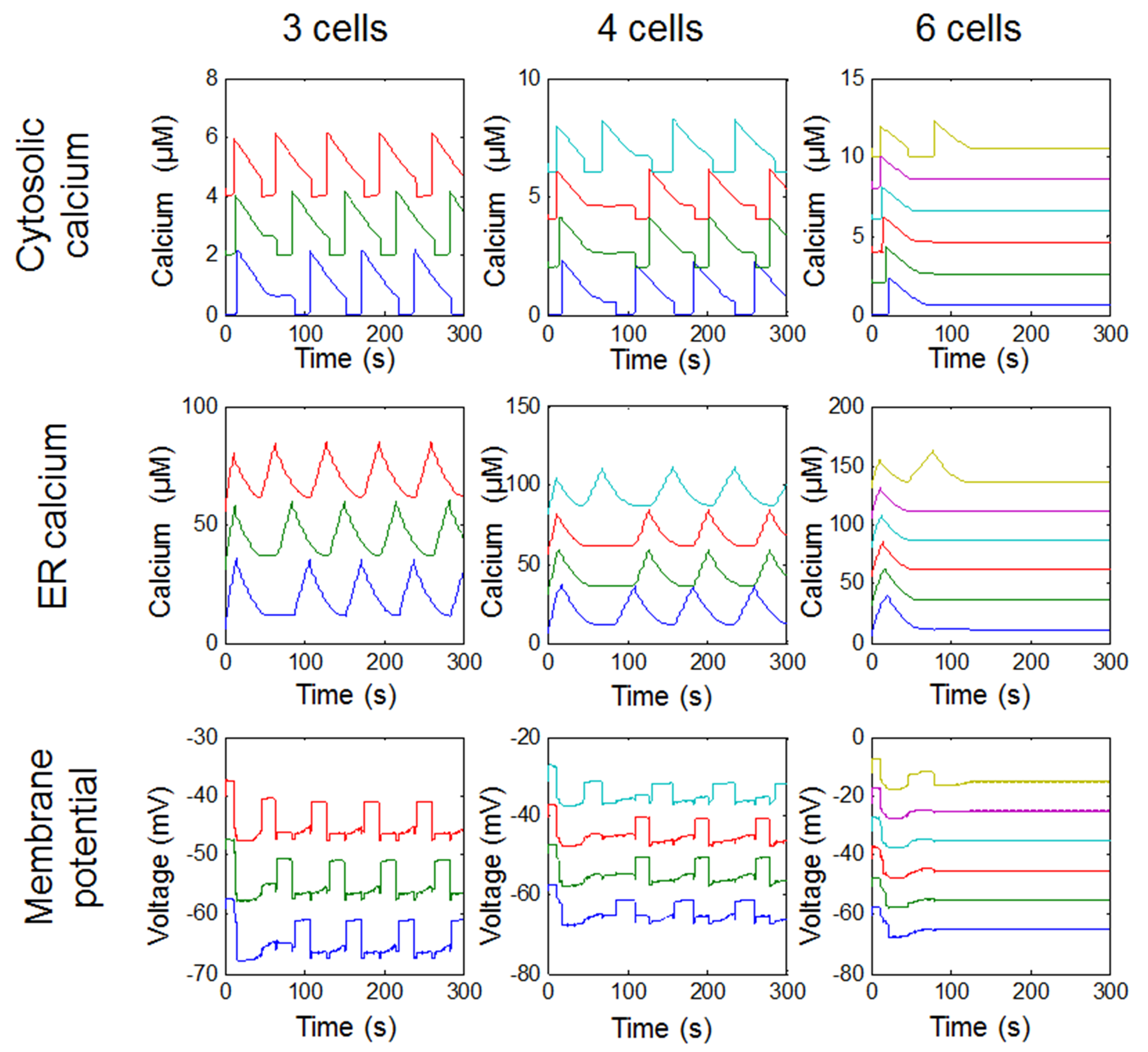

Supplement: S7 Fig — Effects of the number of coupled cells on cytosolic calcium, ER calcium, and membrane potential in 3, 4, and 6 cells connected. The data were shifted vertically for clarity. (TIF) [file pcbi.1004955.s007.tif]

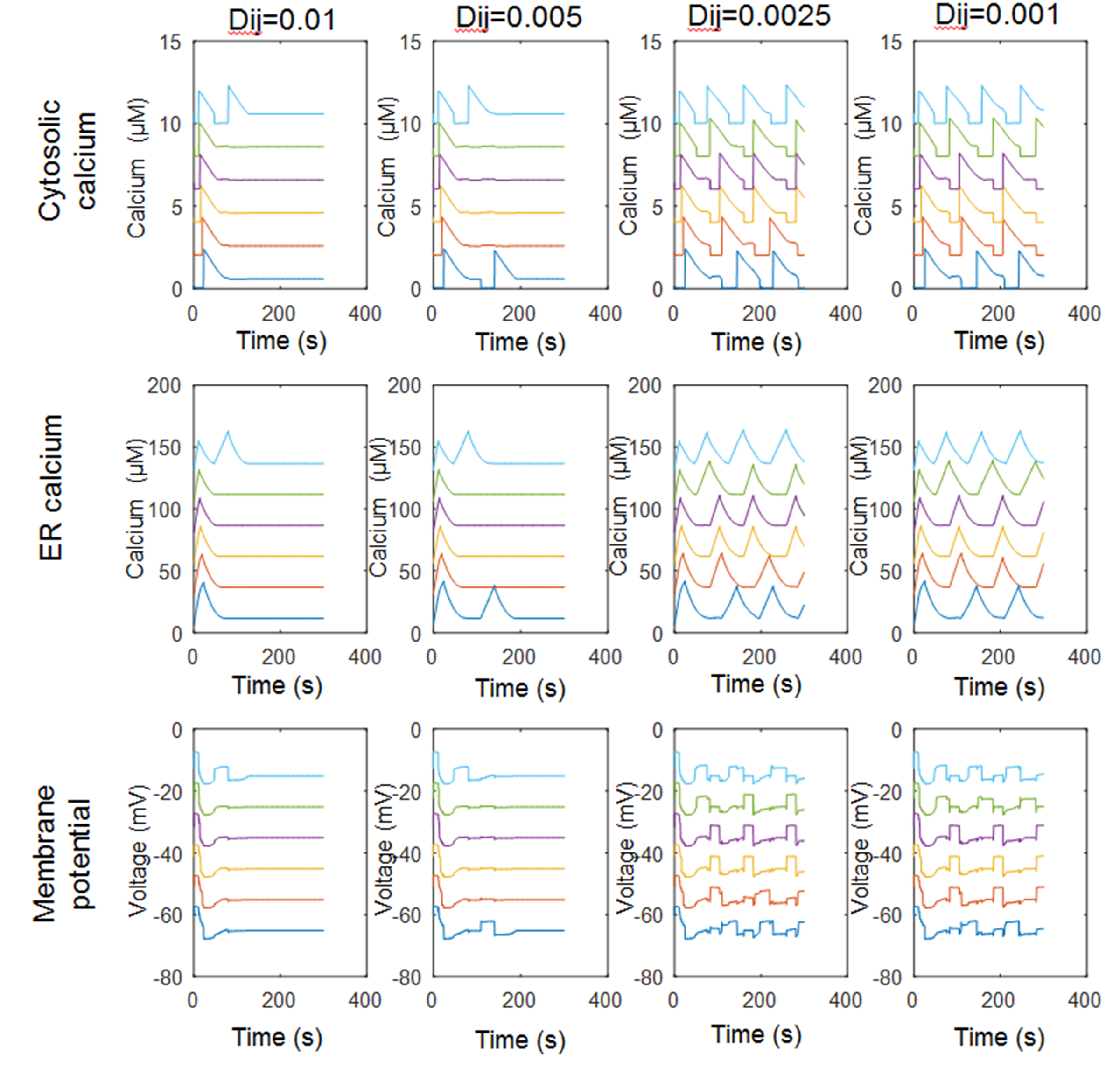

Supplement: S8 Fig — Effects of the calcium diffusivity of coupled cells on cytosolic calcium, ER calcium, and membrane potential. The data were shifted vertically for clarity. (TIF) [file pcbi.1004955.s008.tif]

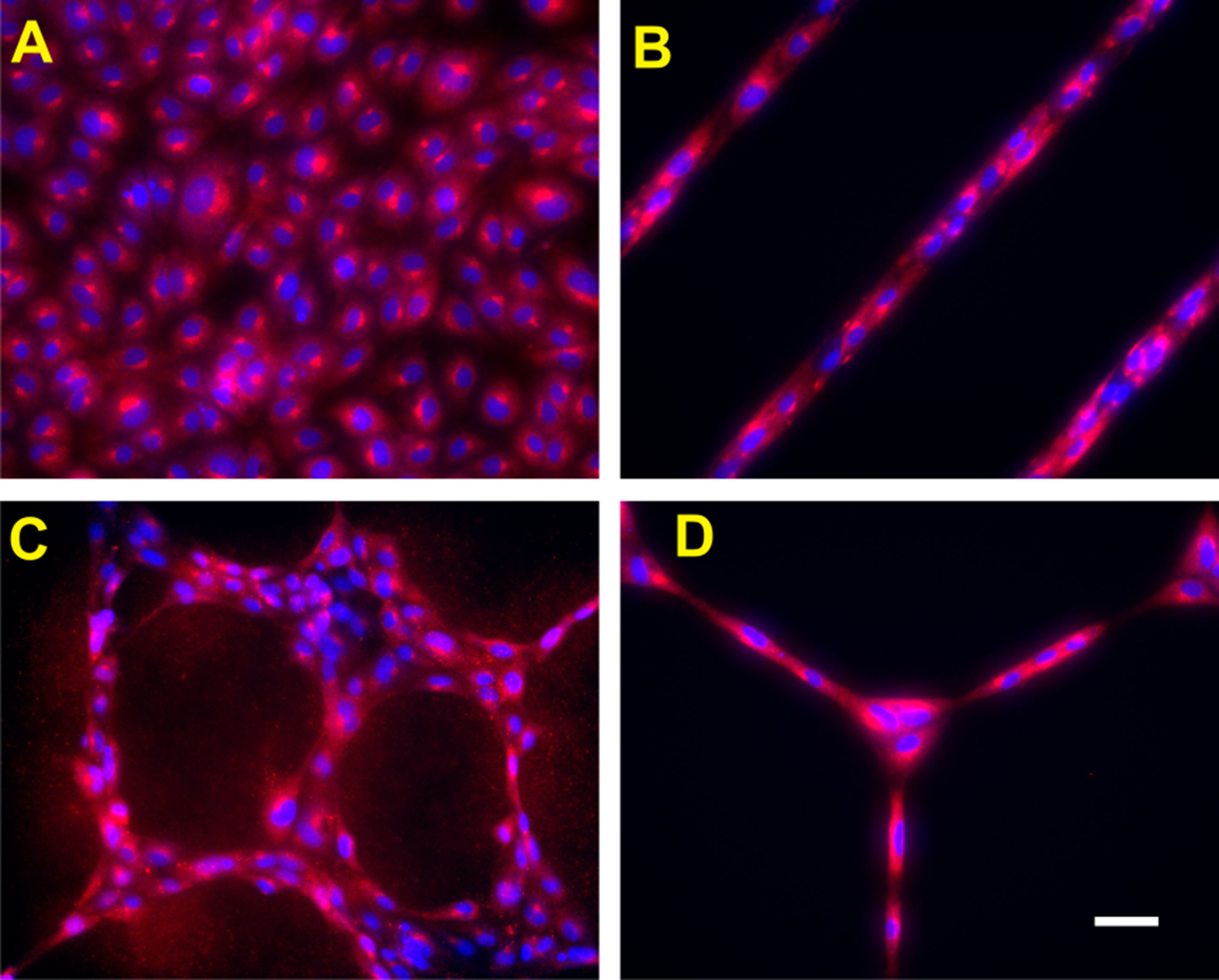

Supplement: S9 Fig — (A-D) Immunofluorescence staining of histamine H1 receptors (red) and nuclei (blue) in (A) monolayers, (B) linear cell networks, (C) capillary-like networks, and (D) hexagonal cell networks. Scale bar, 40 μm. (TIF) [file pcbi.1004955.s009.tif]
