# Supplementary material for: Cellular Architecture Regulates Collective Calcium Signaling and Cell Contractility
Source: PLoS Comput Biol. 2016 May 19;12(5):e1004955. doi: 10.1371/journal.pcbi.1004955 (PMC4873241; doi:10.1371/journal.pcbi.1004955)
Supplement: S1 Text — Computational modeling of pluricellular calcium dynamics. (DOCX) [file pcbi.1004955.s016.docx]

**Supplementary note**

**Computational modeling of pluricellular calcium dynamics**

**Calcium dynamics in single cells.** Cytosolic calcium plays essential roles in the regulation of various biological processes.[^1^](#_ENREF_1) The mechanisms that drive calcium dynamics in cells have been studied extensively. A key feature of intracellular calcium signaling is calcium-induced calcium release. In particular, the release of calcium from intracellular stores, such as the endoplasmic reticulum (ER), is regulated by cytosolic calcium itself through multiple positive and negative feedback loops. The cytosolic calcium concentration can also be controlled by the efflux of calcium and the voltage-dependent calcium current depending on the cell type.

Several types of computational models have been developed for studying cellular calcium dynamics.[^2^](#_ENREF_2) These models differ from each other by the underlying assumptions and the implementation (e.g., deterministic, threshold, and stochastic) of the calcium signaling mechanisms. In this study, we establish a computational model to study the effects of cellular architecture in collective calcium signaling based on minimal models that effectively capture the calcium signaling dynamics.[^3-5^](#_ENREF_3) S1-2 equations describe the calcium concentrations in the cytosol, *C_c_*, and ER, *C_e_*.

$\frac{dC_{c}}{dt}=J\left( C_{c},C_{e} \right)-KC_{c}-\Phi I_{Ca}$ (S1)

$\frac{dC_{e}}{dt}=-J(C_{c},C_{e})$ (S2)

*J(C_c_,C_e_)* depicts the calcium flux between the cytosol and ER (S3 equation). The amplitude and direction of the calcium flux depend on both cytosolic and ER calcium concentrations. The mathematical function in S3 equation describes the biphasic regulation of inositol trisphosphate receptors by cytosolic calcium and effectively implements calcium-induced calcium release in cells.

$J\left( C_{c},C_{e} \right)=-V_{M2}\frac{C_{c}^{2}}{K_{2}^{2}+C_{c}^{2}}+\left( V_{M3}\frac{{(K_{4}C_{c})}^{3}}{{({C_{c}+K}_{4})}^{6}}+K_{s} \right)C_{e}$ (S3)

The second term of S1 equation (*KC_c_*) models the efflux of calcium. The third term of S1 equation represents the calcium current that is dynamically interacting with the membrane potential, *V* (S4-7 equations).

$C\frac{dV}{dt}=-I_{Ca}-I_{K\_Ca}-I_{leak}$ (S4)

$I_{Ca}=g_{Ca}{(\frac{1}{1+e^{{-(V-V_{m})}/{T_{m}}}})}^{3}(\frac{1}{1+e^{{V-V_{h}}/{Th}}})\left( V-V_{Ca} \right)$ (S5)

$I_{K\_Ca}=\frac{g_{K_{Ca}}}{2}\left\{ 1+tanh(\beta\left[ C_{c}-X^{*} \right]) \right\}\left( V-V_{K} \right)$ (S6)

$I_{leak}=g_{leak}(V-V_{leak})$ (S7)

These equations capture the major calcium processing mechanisms and are capable of generating calcium pulses and calcium-induced calcium release, which are major features of calcium dynamics.

**Model parameters**

The model parameters were obtained from previous studies (Table S1).[^3^](#_ENREF_3)^,^ [^6^](#_ENREF_6) In general, the calcium dynamics is cell type specific and depends on the agonist concentration. We, therefore, performed experiments to measure the calcium dynamics in individual endothelial cells under 0.5 µM, 1 µM, 2 µM and 5 µM of histamine (Fig. 3 of main text). In the experiment, calcium oscillations were observed when the histamine concentration was below 3 µM. The calcium pulse typically had a duration between 20 seconds and 50 seconds. The interval between the calcium pulses was widely distributed from 20 seconds to over 200 seconds (Fig. 3B-D of main text). The interval between calcium pulses decreased slightly with the histamine concentration while the amplitude of the calcium pulses remained constant at low histamine concentrations. The calcium oscillation occurrence rate decreased with the histamine concentration. Consistent with previous studies,[^7^](#_ENREF_7) less than 10% of cells exhibited calcium oscillation at 5 μM.

The cytosolic calcium dynamics depends on the calcium fluxes from the extracellular space and the endoplasmic reticulum. The experimental data were applied to fit the model parameters. To evaluate the ability of the model to represent the calcium dynamics under different histamine concentrations, the rates of calcium flux, i.e., *J(C_c_,C_e_)*, to the extracellular space and the endoplasmic reticulum were adjusted systematically. Adjusting the calcium flux modulated the calcium waveform and the duration of calcium pulses (Fig. 3I-L of main text). As shown in Fig. 3, the model was capable of capturing the calcium waveforms (duration and interval between pulses) and oscillatory behaviors observed under different histamine concentrations. In this study, *K* = 0.5 s^-1^ and *V_m3_* = 500 s^-1^ were chosen to mimic the behaviors of cells treated with 5 μM histamine.

**Table S1.** Parameters in the computational model of calcium signaling.

| Symbols | Values | Units | Symbols | Values | Units |
| --- | --- | --- | --- | --- | --- |
| *K* | 0.5 | s^-1^ | ***V_m_*** | -61 | mV |
| *K_s_* | 0.01 | s^-1^ | ***T_m_*** | 4.2 | mV |
| *V_m2_* | 100 | µM/s | ***V_h_*** | -85.5 | mV |
| *V_m3_* | 500 | s^-1^ | ***T_h_*** | 8.6 | mV |
| *K_2_* | 0.2 | µM | ***V_Ca_*** | 120 | mV |
| *K_4_* | 0.69 | µM | ***g_Ca_*** | 100 | µS/cm^2^ |
| *φ* | 9.221×10^-3^ | µMcm^2^/(S⋅nA) | ***β*** | 2.5 | µM^-1^ |
| *g_ij_* | 1000 | µS/cm^2^ | ***X**** | 0.4334 | µM |
| *D_ij_* | 0.012 | s^-1^ | ***Vk*** | -85 | mV |
| *V_leak_* | -55 | mV | ***g_K_Ca_*** | 2000 | µS/cm^2^ |
| *g_leak_* | 2701 | µS/cm^2^ | ***C*** | 1 | µF/cm^2^ |

**The behaviors of two coupled cells.** The model was applied to study the effects of gap junctional intercellular communication (GIJC) on collective calcium dynamics. S1-4 equations were replaced with S8-10 equations to incorporate electrical and biochemical couplings between the cells. Electrical coupling leads to coupling currents, *I_coupling_,* between the cells and biochemical coupling results in diffusion of cytosolic calcium via GIJC.

$\frac{dC_{c}^{i}}{dt}=J\left( C_{c}^{i},C_{e}^{i} \right)-KC_{c}^{i}-\Phi I_{Ca}^{i}-D_{ij}\sum(C_{c}^{i}-C_{c}^{j})$ (S8)

$C\frac{dV^{i}}{dt}=-I_{Ca}^{i}-I_{K\_Ca}^{i}-I_{leak}^{i}-I_{coupling}^{i}$ (S9)

$I_{coupling}^{i}=\sum_{j} g_{ij}(V^{i}-V^{j})$ (S10)

To illustrate the effects of electrical and biochemical coupling, a two-cell model was first developed (S3 Fig.). For uncoupled cells, calcium oscillation did not occur. When two cells were electrically coupled with sufficient coupling strength, *g_ij_*, anti-phase calcium oscillations occurred in the cells. Examining the ER calcium and membrane potential revealed the mechanism of calcium oscillation. A large electrical coupling strength can multiply a small voltage difference between the cells to generate a significant current flow (see S10 equation). The coupling current is compensated by other currents (e.g., calcium current) to maintain the membrane potential (S9 equation). The calcium current, in turn, perturbs the cytosolic calcium from the rest state. The electrical coupling results in out of phase oscillations in cytosolic calcium, ER calcium and membrane potential between the cells (S3A Fig.).

The influence of the coupling strength was studied systematically (S3B Fig.). For two coupled cells, no oscillations were observed with weakly coupled cells (*g_ij_* less than 500). With a sufficiently large coupling strength, the perturbation due to the coupling current destabilizes the system to trigger calcium oscillations. The influence of the effective diffusivity of calcium was also investigated. In contrast to electrical coupling, the biochemical coupling stabilizes the system and increases the threshold of coupling strength for calcium oscillations to occur (S3B Fig.). Furthermore, the effects of electrical and biochemical coupling depend on the number of couple cells, resulting in the architecture dependence observed in this study (S7-8 Fig. and Fig. 4 in main text).

**Supplementary reference**

1. Clapham DE. Calcium signaling. *Cell*. 2007;131:1047-1058

2. Dupont G, Combettes L, Bird GS, Putney JW. Calcium oscillations. *Cold Spring Harb Perspect Biol*. 2011;3

3. Loewenstein Y, Yarom Y, Sompolinsky H. The generation of oscillations in networks of electrically coupled cells. *Proc Natl Acad Sci U S A*. 2001;98:8095-8100

4. Goldbeter A, Dupont G, Berridge MJ. Minimal model for signal-induced ca2+ oscillations and for their frequency encoding through protein phosphorylation. *Proc Natl Acad Sci U S A*. 1990;87:1461-1465

5. Cornelisse LN, Scheenen WJJM, Koopman WJH, Roubos EW, Gielen SCAM. Minimal model for intracellular calcium oscillations and electrical bursting in melanotrope cells of xenopus laevis. *Neural Computation*. 2001;13:113-137

6. Shen P, Larter R. Chaos in intracellular ca2+ oscillations in a new model for non-excitable cells. *Cell Calcium*. 1995;17:225-232

7. Jacob R, Merritt JE, Hallam TJ, Rink TJ. Repetitive spikes in cytoplasmic calcium evoked by histamine in human endothelial cells. *Nature*. 1988;335:40-45
